# The Role of Long Non-Coding RNAs in Hepatocarcinogenesis

**DOI:** 10.3390/ijms19030682

**Published:** 2018-02-28

**Authors:** Manuela Lanzafame, Gaia Bianco, Luigi M. Terracciano, Charlotte K. Y. Ng, Salvatore Piscuoglio

**Affiliations:** 1 Institute of Pathology, University Hospital Basel, Basel 4031, Switzerland; manuela.lanzafame@usb.ch (M.L.); gaia.bianco@usb.ch (G.B.); luigi.terracciano@usb.ch (L.M.T.); kiuyancharlotte.ng@usb.ch (C.K.Y.N.); 2 Department of Biomedicine, University of Basel, Basel, 4056 Switzerland

**Keywords:** long non-coding RNA, hepatocellular carcinoma, liver cancer, carcinogenesis

## Abstract

Whole-transcriptome analyses have revealed that a large proportion of the human genome is transcribed in non-protein-coding transcripts, designated as long non-coding RNAs (lncRNAs). Rather than being “transcriptional noise”, increasing evidence indicates that lncRNAs are key players in the regulation of many biological processes, including transcription, post-translational modification and inhibition and chromatin remodeling. Indeed, lncRNAs are widely dysregulated in human cancers, including hepatocellular carcinoma (HCC). Functional studies are beginning to provide insights into the role of oncogenic and tumor suppressive lncRNAs in the regulation of cell proliferation and motility, as well as oncogenic and metastatic potential in HCC. A better understanding of the molecular mechanisms and the complex network of interactions in which lncRNAs are involved could reveal novel diagnostic and prognostic biomarkers. Crucially, it may provide novel therapeutic opportunities to add to the currently limited number of therapeutic options for HCC patients. In this review, we summarize the current status of the field, with a focus on the best characterized dysregulated lncRNAs in HCC.

## 1. Introduction

Hepatocellular carcinoma (HCC) is one of the several malignancies in which mortality has been increasing, in particular, in Western populations [1]. HCCs typically arise on a background of cirrhosis and are usually associated with chronic hepatitis B (HBV) or hepatitis C virus (HCV) infection, exposure to aflatoxin B1, alcoholic liver disease, obesity or metabolic disorders. Treatment options for early-stage HCC involve resection and/or liver transplantation. On the other hand, for late-stage patients, only three systemic agents, namely sorafenib [2], regorafenib (both kinase inhibitors [3]), and nivolumab (an immune checkpoint inhibitor) [4], have been approved. Novel therapeutic targets may improve the dismal prognosis of late-stage HCC patients.

An increasing body of evidence suggests that the non-coding regions of DNA play fundamental roles in the regulation of many biological processes, including physiological processes such as cell growth and cell proliferation [5], cell migration [6], metabolism, and apoptosis [7]. Indeed, it is now believed that ~70–80% of the human genome does not encode for proteins but is transcribed as non-coding RNA (ncRNA) molecules [8,9]. A substantial portion of this “genomic dark matter” is long non-coding RNA (lncRNA), defined as ncRNA greater than ~200 nucleotides in length, accounting for 68% of RNA molecules [10]. lncRNA transcripts share some of the properties of protein-coding transcripts. For instance, lncRNA transcription is regulated by histone modification and canonical spliceosome machinery [11,12,13]. Mechanistically, lncRNAs interact with transcription factors and may variably guide them to [14] or prevent them from binding to their target genes [15]. They may also act as enhancers by rearranging chromatin or may act as sponges to bind proteins or microRNAs [16,17,18,19]. Of their many roles, the best described is the recruitment of chromatin modifying complexes to specific genomic regions [6,11,13,20,21,22,23] via chromosomal looping [24,25].

Given their multiple functions in transcriptional, post-transcriptional, and epigenetic regulation of gene expression, they are emerging as new players in tumorigenesis. Indeed, increasing evidence demonstrates that dysregulation of lncRNAs is involved in several pathological conditions, including various types of cancer, such as those of the breasts, the lungs, prostate, and liver [26]. Two pan-cancer studies using The Cancer Genome Atlas (TCGA) data have revealed that lncRNA expression is tissue-, cell-type- and cancer-specific [10,27]. Of these two studies, one described 7942 lineage- or cancer-associated lncRNAs across cancer types [10], while the other found that 60% of the dysregulated lncRNAs is cancer type-specific [27].

Although there have been an increasing number of studies on lncRNAs in the past decade, the contribution of lncRNAs to HCC development, metastasis, and recurrence remains largely unknown. In the last years, many studies have been directed to the discovery and characterization of the contribution of lncRNAs to carcinogenesis and their potential use as diagnostic markers or therapeutic targets for HCC treatment. In this review, we will describe the biological roles of lncRNAs, their mechanisms of action, and the lncRNAs dysregulated in HCC. We will further provide an overview of the latest studies that are aimed at elucidating the potential uses of lncRNAs as diagnostic/prognostic markers and as therapeutic targets in HCC.

## 2. LncRNAs: Characteristics and Subclassification

Among the many subclasses of ncRNA molecules, lncRNAs are defined as non-coding transcripts that are more than ~200 nucleotides in length [28]. The most comprehensive characterization of this class of non-coding RNA was carried out by the GENCODE consortium, which reported on the extensive annotation of 14,880 human lncRNA species [12]. LncRNAs share many common features with mRNA transcripts; they are both transcribed by RNA polymerase II, they both undergo splice-processing and post-transcriptional modifications (5′-capping and polyadenylation), and they share similar chromatin states. Unlike mRNAs, lncRNAs tend to be shorter, are expressed at lower levels, and display fewer but longer exons [29]. Although lncRNAs show poor sequence conservation among species, their peculiar secondary structures, mechanisms of action, and localization appear to be highly conserved [30]. These features allow for lncRNAs to be classified according to several divergent criteria [31]. lncRNA subcellular localization, for instance, provides valuable information related to their functions and mechanisms of action. The known subcellular localization of lncRNAs based on RNA sequencing data has been collated into the lncATLAS database [32]. In general, lncRNAs tend to be more abundant in the nucleus [31], with some of them being reported to be chromatin-associated RNAs (CARs) [23] and some others that are directly related to the formation of nuclear bodies, such as *NEAT1* and *MALAT1* [33,34]. Single molecule RNA FISH analysis, revealed that even nuclear localization can be further categorized in distinct nuclear patterns [35], with the presence of both bright nuclear foci with distinct lncRNAs and single dispersed nuclear lncRNAs. Of note, some lncRNAs have been reported to be enriched in the cytosol and to localize with ribosomes [36]. Interestingly, lncRNAs have also been reported to be encoded in the small mitochondrial genome [37].

**Figure 1:**
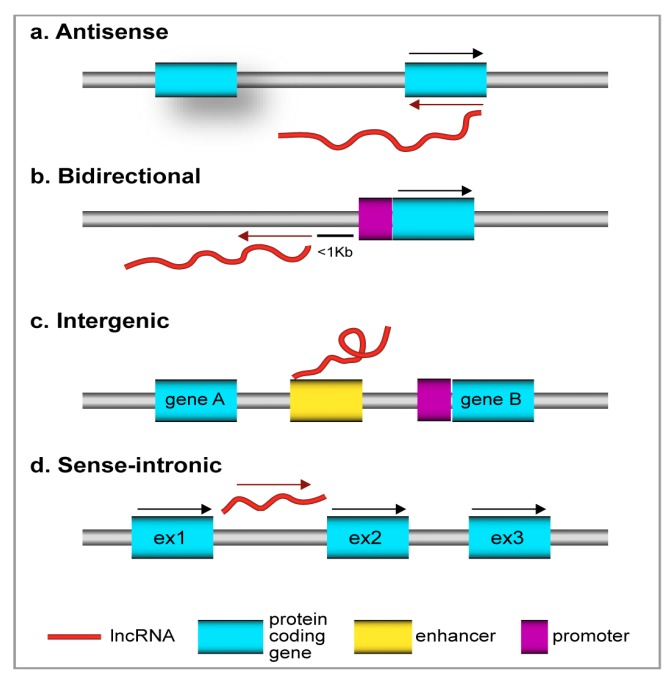
Long non-coding RNA (LncRNA) classification in the context of different genomic locations. On the basis of genomic location and orientation to genes there are four major classes of lncRNAs. (**a**) Antisense lncRNAs are transcribed from the opposite strand of coding genes; (**b**) Bidirectional lncRNAs are transcribed from the opposite strand, in the opposite direction and within 1 kb of the promoter of coding genes; (**c**) Intergenic lncRNAs are transcribed in the genomic region between two coding genes and usually are located in enhancer regions acting in *cis* on the promoters of the downstream genes; and, (**d**) Sense-intronic lncRNAs are transcribed from the sense strand of an intronic region with no overlap of exonic sequence. Ex: exon.

Despite the diverse features that are displayed by lncRNAs, as a general rule, they are broadly classified according to their biogenesis and genomic positions in relation to protein-coding genes, lncRNAs can be broadly classified into: (i) antisense RNAs or natural antisense transcripts (NATs); (ii) bidirectional RNAs; (iii) long intergenic RNAs (lincRNAs); and (iv) sense intronic RNAs [12,38,39] (Figure 1).

### 2.1. Antisense RNAs or Natural Antisense Transcripts (NATs)

NATs are endogenous RNAs that partially or totally overlap transcripts originating from their opposite strand. Yelin et al., estimated that more than 8% of the predicted 40,000 human genes have an antisense partner [40]. A substantial portion of eukaryotic promoters may indeed be transcribed in both the sense and the antisense directions [41]. Sense and antisense transcripts are usually regulated in a coordinated way, such as that high levels of the sense transcript usually lead to high levels of the antisense transcript, and vice versa.

### 2.2. Bidirectional RNAs

Bidirectional RNAs are also known as promoter-associated non-coding RNAs (pancRNAs) [42]. Slightly different from NATs, bidirectional RNAs are transcribed in the opposite direction with respect to the protein coding gene, but are located within 1 kb from its promoter region [42,43,44,45]. An example of this class of lncRNA is *Linc00441*, the bidirectional transcribed lncRNA of the Retinoblastoma gene *RB1*. *Linc00441* has recently been reported to be aberrantly upregulated and inversely correlated to *RB1* expression in human HCC samples [46]. More specifically, *Linc00441* has also been reported to epigenetically suppress *RB1* expression in HCC by recruiting DNMT3A methyltransferase [46].

### 2.3. Long Intergenic RNAs (lincRNA)

LincRNA refers to the class of non-coding RNAs that are transcribed from intergenic regions between two protein-coding genes [47]. The majority of lincRNAs are enhancer RNAs (eRNAs) that are located in enhancer regions and usually act in *cis* by inducing chromatin modifications in the promoters of the downstream genes [24]. A classic example of eRNA is the HOXA transcript at the distal tip (*HOTTIP*), a lncRNA situated in the 5′ distal region of the *HOXA* locus [25]. As one of the best characterized lncRNAs implicated in HCC, *HOTTIP* will be extensively described in one of the following sections.

### 2.4. Sense Intronic RNAs

Sense intronic RNAs are transcribed from the introns of protein-coding genes and do not overlap exonic sequences. Examples of sense intronic RNAs are small nuclear-lncRNAs (sno-lncRNAs) and circular intronic lncRNAs (circRNAs). Sno-lncRNAs are lncRNAs that are flanked by two small nuclear RNAs (sno-RNAs) and thereby lack any 5′ and 3′ processing [48]. CircRNAs, instead, form a peculiar class of lncRNA that undergoes a special splicing (back-splicing) thus resulting in chemically circularized molecules [49].

## 3. LncRNAs: Mechanisms of Action

**Figure 2:**
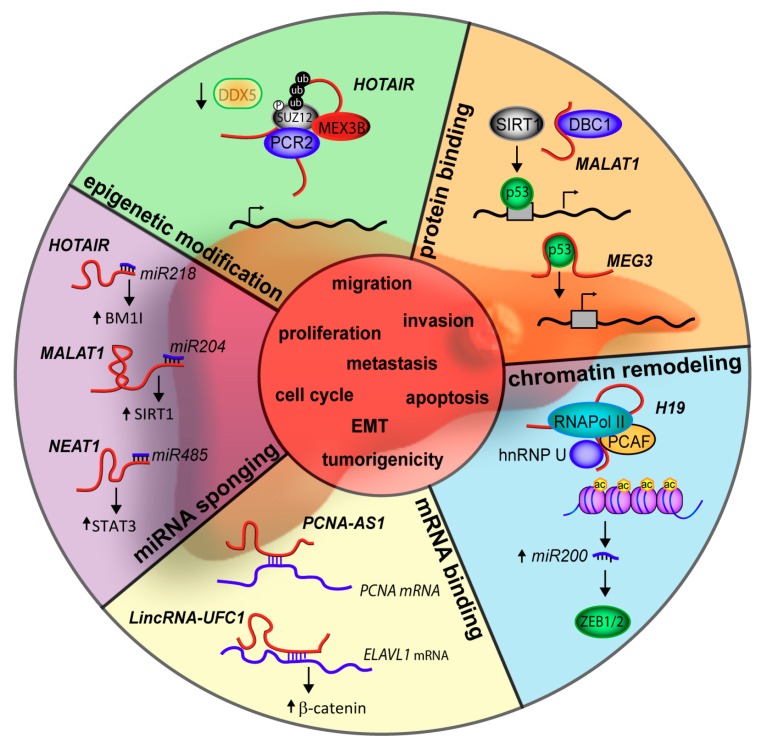
Mechanism of function of lncRNAs dysregulated in hepatocellular carcinoma (HCC). LncRNAs may act as sponges for miRNAs, bind transcripts or proteins and induce epigenetic modifications or chromatin remodeling. Their deregulation leads to hepatocellular carcinogenesis by regulating different cellular processes such as migration, proliferation, invasion, cell cycle, apoptosis and epithelial mesenchymal transition (EMT). Examples of the mechanisms of action of lncRNAs dysregulated in HCC are reported. See text for details.

RNAs are very versatile molecules; they can interact with other nucleic acid molecules by simple base pair coupling and they can interact with proteins by folding into three-dimensional (3D) structures and generating complex recognition surfaces. RNAs are also dynamic; they can be both transcribed and degraded rapidly [50]. The versatility and dynamic nature are particularly evident for lncRNAs whose ability to bind both nucleic acids and proteins enables them to regulate gene expression on the transcriptional, post-transcriptional, and protein levels (Figure 2).

### 3.1. Transcriptional Regulation and Chromatin Modification

One of the best described mechanisms of action of lncRNAs is their capacity to induce epigenetic modifications by acting as a scaffold for chromatin modification complexes. This discovery provided insight into the previously unresolved question on how chromatin modification complexes are able to act on the whole genome in a time-dependent and a cell-specific manner. Indeed, lncRNAs can guide chromatin remodeling complexes to specific genomic regions, regulating gene expression either in *cis* or in *trans*, as enhancers and mediators for long-range chromatin interactions [20,21].

The role of lncRNA as a scaffold for chromatin remodeling complexes was first described for the HOX transcript antisense RNA (*HOTAIR*). *HOTAIR* was reported to interact with the Polycomb repressive complex 2 (PRC2), thereby repressing the expression of the *HOXD* gene locus by inducing histone methylation and heterochromatin formation [51]. The same mechanism has now been demonstrated for other lncRNAs, such as *XIST* [52], the lncRNA that is responsible for X-chromosome inactivation, and it appears to be a general mechanism by which lncRNAs regulate gene expression during imprinting, development, cell differentiation, and disease [53,54].

Chromatin modification is not the only way through which lncRNAs modulate transcription. NATs, for instance, are able to directly inhibit the transcription of their sense transcripts in *cis* by competing for RNA polymerase II [55] or by forming an RNA-DNA triplex that prevents the binding of the transcription initiation complex [15]. Additionally, lncRNAs can fold into secondary structures that mimic DNA binding sites, further inhibiting the nuclear export of transcriptional factors by directly interacting with and repressing their associated transport proteins [56].

### 3.2. Post-Transcriptional Regulation and Maintenance of mRNA Stability

On the post-transcriptional level, lncRNAs play a role in regulating mRNA splicing. For example, NATs can form RNA-RNA duplexes which can mask splice sites [57]. Other lncRNAs, such as the metastasis-associated lung adenocarcinoma transcript 1 (*MALAT1*), have been reported to regulate splicing by directly modulating the activity of Ser/Arg-domain rich splicing factors [58].

LncRNAs may also influence mRNA stability by acting as competing endogenous RNAs (ceRNAs) [59,60,61] that compete for the binding of shared miRNAs. In particular, the circRNA subclass almost exclusively acts as miRNA decoy [17]. An example is *circMTO1* that has been recently described to promote HCC cell proliferation and invasion by binding *miR-9* and down-regulating p21 [62]. In general, miRNA sponging is a common mechanism among lncRNAs, owing to the presence of miRNAs competing target sites in their sequences. When the lncRNA with the complementary sequence becomes transcriptionally active, it competes for miRNA targeting and binding of RISC complexes, thus resulting in increased parent gene expression [18]. Several examples have been reported in HCC, such as the lncRNA highly upregulated in liver cancer (*HULC*) [16], *MALAT1* [63], and nuclear enriched abundant transcript 1 (*NEAT1*) [64]. LncRNAs may also help to destabilize mRNA transcripts by activating a specific type of mRNA decay, called Staufen-mediated decay. This is the case for *Alu* repeats-containing lncRNAs, which can bind *Alu* sequences in the 3’UTR of target genes, thus mediating the binding of Staufen1 and the subsequent mRNA degradation [65].

### 3.3. Protein Activity Regulation and Scaffolding

Several lncRNAs have been reported to regulate cellular processes by direct binding to proteins, including RNA-binding proteins (RBPs) and transcription factors [66]. For example, *Gadd7* and *MALAT1* bind to and modulate the expression of RBP TDP43 [67], which is a protein that is implicated in mRNA splicing, transport, and stability [68]. Moreover, in silico prediction suggested that the HCC-associated lncRNAs *HOTTIP*, *H19*, *HOTAIR*, *MALAT1*, *AIRN*, *MEG3,* and *uc002mb* may interact with the RBPs eIF4AIII, PTB, and FUS [69]. In addition, lncRNAs harbor several distinct domains, each able to bind to distinct effector molecules, thus enabling them to serve as adaptors to bring proteins into complexes [66]. Apart from the role that lncRNAs play in chromatin remodeling, they may also serve as scaffolds for nuclear domains [70]. One such example is *NEAT1*, which has been implicated in the de novo assembly of the subnuclear organelles paraspeckles [71].

## 4. Widespread lncRNA Dysregulation in HCC

Significant effort has been made to examine the expression of lncRNAs and its relationship with carcinogenesis. The use of lncRNA microarrays and next generation sequencing techniques has allowed for researchers to perform genome-wide analyses to identify a large number of lncRNAs aberrantly expressed in HCC tissue and may be involved in hepatocarcinogenesis.

Cui et al. [72] performed a comprehensive investigation into lncRNA expression profile in HCC and matched non-tumor counterpart using two RNA sequencing datasets (including that of TCGA), and two lncRNA microarray datasets. The authors identified 347 lncRNAs that were consistently up- or down-regulated in at least two datasets [72]. They found that 31 and 41 lncRNA loci were located in genomic regions with recurrent DNA gains or losses, respectively, suggesting that genomic copy number alterations may be involved in the dysregulation of some lncRNAs in HCC. Furthermore, by comparing lncRNA expression pattern in HCC with or without invasion and metastasis, they also identified lncRNAs that may be involved in cancer cell metastasis and HCC recurrence [72].

Similar genome-wide lncRNA profiling studies carried out by other groups have invariably pointed to the general conclusion of widespread lncRNA dysregulation in HCC. An RNA-sequencing study of HBV-related HCC samples revealed a total of 1242 dysregulated lncRNA transcripts (983 up-regulated and 259 down-regulated) [73]. In another study of 12 HCC tissues and paired adjacent normal tissues, the authors identified 214 differentially expressed lncRNAs, among which 17 were further confirmed in 21 paired HCC and normal liver tissues via quantitative real-time PCR [74]. Finally, an RNA-sequencing analysis from 20 HCC patients recently identified 8603 novel dysregulated lncRNAs, including 917 recurrently dysregulated lncRNAs that were associated with clinicopathologic features [75]. Of particular interest was the observation that approximately 76% of the HCC-related lncRNAs were not previously annotated by the MiTranscriptome [10] or GENCODE [76], suggesting that at least some of the dysregulated lncRNAs might be HCC-specific [75].

### 4.1. Molecular and Functional Alterations of lncRNAs in HCC

Despite the huge number of lncRNAs described to be dysregulated in HCC by genome-wide approaches, not many have been comprehensively characterized (Table 1 and S1).

The vast majority of them is reported to be up-regulated in HCC and have been shown to have an oncogenic effect by promoting cell proliferation, invasion, metastasis formation, and/or angiogenesis. Investigations into the expression of these oncogenic lncRNAs have found them to be positively correlated with clinicopathological features of the patients. In this section, we will describe some of the well-characterized lncRNAs dysregulated in HCC in detail, giving some examples of their molecular mechanisms in cancer and their specific role in hepatocellular carcinogenesis. An extended list of the lncRNAs, their dysregulation, and molecular functions in HCC can be found in Table S1.

#### 4.1.1. HOTAIR

One of the most well studied lncRNAs in HCC is the *HOX* transcript antisense intergenic RNA (*HOTAIR*) identified from a custom tilling array of the *HOXC* locus (12q13.13). *HOTAIR* represses transcription in *trans* acting as a scaffold for at least two distinct histone modification complexes to the target gene promoters: PRC2 and the lysine-specific demethylase 1 (LSD1)/co-repressor of RE1-silencing transcription factor (coREST)/REST complex [21,51,117].

Several studies showed that *HOTAIR* levels are elevated in HCC [82,83,84,85,86,87], and that its expression is associated with patients with increased risk of recurrence or metastasis [82,86], poor prognosis [84] and significantly shorter recurrence-free survival [83]. At the cellular level, *HOTAIR* is involved in proliferation, cell motility, viability and invasion, cell cycle progression, apoptosis, autophagy, and chemotherapeutic sensitivity of cancer cells to cisplatin and doxorubicin [83,84,85,86,87,88,117,118,119,120].

Recently, several studies have revealed novel insights into the functional mechanisms of *HOTAIR* in HCC cells. Su et al., demonstrated that *HOTAIR* expression is regulated by FOXC1 and that the oncogenic activity of *HOTAIR* is in part based on its sponging of *miR-1* [85]. Indeed, miRNA sponging appears to be a general mechanism of *HOTAIR*, as *HOTAIR* also targets *miR-218*, resulting in increased cell viability, cell cycle upregulation, and tumorigenicity [120]. In primary human HCC specimens, *HOTAIR* was shown to be concordantly upregulated with the oncogene *BMI1*, which is a target of *miR-218*. Interestingly, *HOTAIR* silencing activates the main *BMI1* downstream targets P16(Ink4a) and P14(ARF), by enhancing *miR-218* and inhibiting *BMI1* expression, thus resulting in the suppression of tumorigenesis in HCC [120].

*HOTAIR* also binds to transcripts and proteins. An integrated transcriptomic and quantitative proteomic analysis revealed that a total of 673 transcripts and 293 proteins are regulated by *HOTAIR* [121]. The analysis also showed that *HOTAIR* controls cell proliferation by regulating opioid growth factor receptor (*OGFR*) expression [121]. Cell proliferation in HCC could also be driven by *HOTAIR*-dependent regulation of cell cycle via STAT3 signaling [119]. Besides cell proliferation, *HOTAIR* has also been shown to be involved in the regulation of pluripotency. In fact, the binding of *HOTAIR* with the DDX5-PRC2 complex in the HBx-expressing hepatocytes 4pX-1 regulates the transcription of the epithelial cell adhesion marker EpCAM and pluripotency genes *Nanog*, *Oct4*, and *Sox2* [122]. Emerging evidence also suggests a novel relationship between *HOTAIR* and glucose metabolism in HCC cells by upregulating glucose transporter isoform 1 (*GLUT1*) and activating the mammalian target of rapamycin (mTOR) signaling pathway [87].

#### 4.1.2. HOTTIP

Among the lncRNAs dysregulated in HCC, *HOTTIP* deserves a special mention. This 3764 nucleotide RNA molecule is transcribed 330 base pairs upstream of the *HOXA* locus on chromosome 7p15.2. It could be considered a classical eRNA acting in *cis*. Mechanistically, it is a clear example of an lncRNA that serves as a scaffold for chromatin remodeling complexes. Wang et al. [25] showed that, similar to *HOXA* genes, *HOTTIP* is normally expressed at a very low level in distal/posterior anatomical sites and it is implicated in the transcriptional regulation of the *HOXA* locus. Briefly, they demonstrated that in distal fibroblast (foreskin) cells, *HOTTIP* binds to the WDR5-MLL complex and is able to position the complex in close proximity to the downstream *HOXA* genes by chromosomal looping, thus inducing their H3K4me3 and H3K4me4 methylation and subsequent transcriptional activation [25].

*HOTTIP* has been found dysregulated in several types of cancers. Its role in human carcinogenesis was firstly revealed in HCC [89] and has since been described in colorectal [123,124], gastric [125,126,127], pancreatic [128,129], and lung cancers [130], as well as in osteosarcoma [131] and glioma [132]. The results from these studies were collectively analyzed in three parallel meta-analyses, all of which clearly showed that the high expression of *HOTTIP* correlates with shorter overall survival, higher tumor grade and poor prognosis [133,134,135].

Quagliata et al. [89] reported the upregulation of the *HOTTIP* transcript in 52 snap-frozen HCC biopsies from therapy naive patients. The authors reported a higher expression of *HOTTIP* in non-neoplastic liver disease (excluding steatosis) when compared to normal tissue. For the first time, *HOTTIP* expression was found to be correlated with tumor progression and metastasis formation, as well as with overall patient survival, thus proposing *HOTTIP* as a possible prognostic factor for HCC [89]. Furthermore, the study showed that *HOTTIP* positively regulates *HOXA13* expression in HCC cell lines and that its upregulation induces proliferation in vitro. Similarly, Tsang et al., also reported increased *HOTTIP* expression in HCC, highlighting the progressive upregulation of *HOTTIP* from cirrhotic tissue to pre-neoplastic lesion to early stage HCC [90]. They further confirmed the oncogenic potential of *HOTTIP* in a mouse xenograft model and reported that *HOTTIP* can be regulated by miRNA, specifically by *miR-125b* [90].

The role of miRNAs in *HOTTIP* regulation has been further unraveled by Ge et al., who observed a negative correlation between *HOTTIP* and *miR-192/204* in 48 tumor-normal paired liver samples and showed that *HOTTIP* expression can be regulated by *miR-192* and *miR-204* via the canonical Argonaute2 mediated interference (siRNA) [136]. Specifically, the authors showed that *miR-192* and *miR-204* directly suppress *HOTTIP* in vitro and further identified the *GLS1* gene, which plays a critical role in glutaminolysis and tumorigenesis, as a putative downstream target of *HOTTIP*. Ge et al., thus proposed a novel mechanism of action for *HOTTIP*, in which it explicates its oncogenic potential by directly upregulating glutaminolysis in HCC cells and promoting cancer cell proliferation in a time and dose dependent manner [136].

Despite the extensive functional studies, there remains a lot to be discovered in the functional relevance of *HOTTIP* in HCC. For instance, a meta-analysis of 393 HCC from the TCGA study revealed an association between *HOTTIP* expression and the genes that are involved in the PPAR signaling pathway, opening the doors to the further characterization of the role of *HOTTIP* in HCC [137].

#### 4.1.3. MALAT1

*MALAT1* is transcribed from chromosome 11q1 and was originally identified as a prognostic marker for metastasis and patient survival in non-small cell lung carcinoma [99]. Subsequent studies have shown that *MALAT1* is aberrantly up-regulated in various tumor entities [138,139]. Its upregulation promotes tumor growth and metastasis through a variety of mechanisms, including regulating gene expression by recruiting or regulating the level of serine/arginine-rich protein (SR) family members that are involved in alternative splicing [58,140,141] or binding to active genomic sites [34]. High expression of *MALAT1* has been associated with high grade, metastasis, and poor prognosis of cancer patients [141,142].

*MALAT1*, together with *NEAT1*, is one of the few lncRNAs to be described as frequently mutated in HCC leading to the dysregulation of gene expression and regulatory functions [143]. In HCC, Lai et al., reported that *MALAT1* is overexpressed both in vitro and in vivo [100]. Patients with high expression level of *MALAT1*, associated with elevated levels of α-fetoprotein (AFP), have a significantly increased risk of tumor recurrence after liver transplantation [100]. Mechanistically, what is known about *MALAT1* regulation in HCC is that it is transcriptionally regulated by HIF-2α, forming a positive feedback loop involved in the malignant transformation induced by arsenite [144]. It has also been suggested that *MALAT1* could be regulated by the transcription factor, specificity proteins 1 and 3 (Sp1/3) [145].

The mechanisms by which *MALAT1* promotes cell invasion, migration, growth, motility and metastasis in HCC have been shown to be principally related to its ability to bind to miRNAs and function as a sponge, capturing miRNA and regulating their activities. There are at least two *miR-216b* binding sites in *MALAT1* and the HIF-2α-*MALAT1*-*miR-216b* axis regulates multidrug resistance of HCC cells by modulating autophagy [146]. By sponging and competitive binding to *miR-204*, *MALAT1* releases the *miR-204*-mediated suppression of sirtuin 1, which in turn promotes HCC migration and invasion [63]. Furthermore, the sponging of *miR-146b-5p* by *MALAT1* has also been shown to promote tumor growth and metastasis and has been associated with poor survival in HCC patients [147]. Another example is the binding of *miR-143-3p*, which in turn, regulates the tumor suppressor gene *ZEB1* [148]. Recently, *MALAT1* has been found to act as a ceRNA for *miR-195* that is no longer able to suppress its downstream target *EGFR* [149].

A high-throughput strategy by combining RNA pull-down, quantitative proteomics, bioinformatics, and experimental validation has resulted in the identification of interacting protein partners of *MALAT1* in HCC. Indeed, the interactome of *MALAT1* involves ribosomal proteins and proteins critical in RNA processing, gene transcription, protein degradation, and metabolism regulation [150]. The interaction between *MALAT1* and the depleted in breast cancer 1 protein (DBC1) was further validated and characterized, revealing a novel mechanism by which *MALAT1* regulates p53 activity through the interaction with DBC1 [150].

#### 4.1.4. NEAT1

LncRNA nuclear enriched abundant transcript 1 (*NEAT1*) is so called because of its peculiar and exclusive localization in the sub-nuclear compartment paraspeckle [33]. In this compartment, *NEAT1* can modulate gene expression by retaining mRNA molecules in the nucleus and by mRNA editing [151]. Of note, *NEAT1* is genomically in close proximity to *MALAT1* and both are frequently mutated in HCC [143]. *NEAT1* has also been reported to co-localize with *MALAT1* on active chromatin sites where both interact with proteins that are resident in the nuclear bodies [34]. Despite its emerging relevance in the regulation of gene expression, studies on the role of *NEAT1* in human malignancies have remained limited so far. It is known that *NEAT1* is a crucial regulator in several cancers and acts as a pivotal player in tumorigenesis and metastasis of HCC. Guo et al., firstly reported the clinical relevance of *NEAT1* overexpression in HCC tissues and its association with several clinical features such as the number and size of tumor nodes, metastasis formation, TNM stage, vascular invasion, and tumor cell infiltration [109].

The overexpression and relevance of *NEAT1* in HCC tissues and cell lines have been further confirmed in several recent studies aiming to delineate the functional mechanisms of *NEAT1* in HCC pathogenesis [108,110,111]. These studies have shown that *NEAT1* may act both as a miRNA sponge and as a protein-binding competitor. The ability of *NEAT1* in sponging miRNAs was first described by Fang et al. [108], who reported *NEAT1* overexpression in HCC tissues, as well as its negative correlation with *miR-129-5p* expression. They also proposed a mechanism of action involving the *miR-129-5p*, valosin-containing protein (VCP) and IkB axis. Other studies have described similar negative correlations with *miR-613* [111] and *miR-485* [64]. Interestingly, Zhang et al., showed that by acting as ceRNA for *miR-485*, *NEAT1* is indeed able to enhance STAT3 expression in HCC [64].

The function of *NEAT1* is not restricted to miRNA sponging, as it has also been reported to bind to and compete for the assembling of protein complexes. Mang et al., for instance, demonstrated that *NEAT1* forms a protein complex with the splicing factor U2AF65, thus regulating the heterogeneous nuclear ribonucleoprotein hnRNP A2 expression [110]. hnRNP A2 is also an essential splicing factor that promotes cell proliferation and invasion, and correlates with poor outcome in HCC patients. Since hnRNP A2 is normally inhibited by U2AF65, the authors proposed a mechanism by which *NEAT1* may favor HCC development by sequestrating U2AF65 and releasing hnRNP A2 activity [110]. 

Last but not least, two independent studies have associated *NEAT1* with epithelial-to-mesenchymal transition (EMT) [112,152]. In a study using breast cancer tissues and cell lines, Choudhry et al., identified *NEAT1* as a new transcriptional target of HIF-2α and described its ability to induce paraspeckle formation under hypoxic condition [152]. Similarly, Zheng et al., found that the overexpression of HIF-2α upregulates the level of *NEAT1*, thus promoting EMT and metastasis in hepatoma cell lines [112].

#### 4.1.5. H19

*H19* is transcribed from the critical imprinted locus *IGF2/H19* on chromosome 11p15.5 and it was the first lncRNA identified [153]. In most normal adult tissues, only the paternal allele of *IGF2* is expressed, whereas the maternal imprinted allele of *H19* is usually expressed at high levels during embryonic development, but is rapidly repressed in most tissues after childbirth [154]. *H19* is involved in transcription regulation by binding to hnRNP U and disrupting the hnRNP U-actin complex, thus inhibiting the phosphorylation of the RNA Pol II C-terminal domain at Ser5 and consequently preventing RNA Pol II-mediated transcription [155]. Many studies have shown a strong association between *H19* expression and dysregulated imprinting of the *IGF2/H19 locus* with carcinogenesis in several types of cancer, including HCC [156,157,158,159,160].

*H19* is also a ceRNA that acts as a sponge for miRNAs ([161] and references therein). For example, in breast cancer *H19* regulates EMT and mesenchymal-epithelial transition (MET) by differentially acting as a sponge for *miR-200b/b* and *let-7b* [162].

Recently, a new role of the *H19-IGF2* axis in regulating hepatocyte proliferation has been described in mice. It was demonstrated that *H19* and *Igf2* are negatively regulated by PHB1 and CTCF, which cooperatively bind the imprinting control region (ICR) of the *Igf2/H19* locus [79].

Whether *H19* acts as an oncogene or as a tumor suppressor gene is controversial. Zhang et al. [163] demonstrated that *H19*, in association with hnRNP U/PCAF/RNAPol II, activates *miR-200* family by increasing histone acetylation, thus contributing the suppression of EMT and tumor metastasis. Moreover, they showed that *H19* is significantly downregulated in intratumoral (T) HCC tissues compared with peritumoral tissues (L), and that patients with low T/L ratio of *H19* were linked to poor prognosis [163]. *H19* is also a precursor for *miR-675* and both were found downregulated in HCC cells and their downregulation promotes migration and invasion of HCC *via* the AKT/GSK-3beta/Cdc25A signaling pathway [77]. On the contrary, Yang et al., demonstrated that *H19* is overexpressed in HBV-infected patients and is a risk factor for reduced disease-free survival and increased tumor aggressiveness in HCC patients [78]. 

#### 4.1.6. Other lncRNAs Dysregulated in HCC

*Highly up-regulated in liver cancer (HULC)* is a 500 nucleotide lncRNA on chromosome 6p24.3 and it was first identified as one of the most upregulated genes in HCC [93]. It was described to modulate the deregulation of lipid metabolism in HCC through a signaling pathway involving *miR-9*, PPARA, and ACSL1 [96]. It was also shown to promote hepatocarcinogenesis by perturbing the circadian rhythm through upregulating circadian oscillator CLOCK in hepatoma cells [164]. Recently, *HULC* has been described to regulate several signaling pathways by acting as a sponge for miRNAs. For instance, it promotes tumor progression and metastasis through the *miR-200a-3p*/ZEB1 signaling pathway [92] and promotes tumor angiogenesis in liver cancer through *miR-107*/E2F1/SPHK1 signaling [97]. *HULC* also plays an epigenetic role by enhancing the level of ubiquitin-specific peptidase 22 (USP22) and stabilizing the COX2 protein [94]. Finally, together with USP22/Sirt1, *HULC* attenuates the sensitivity of HCC cells to chemotherapeutic agents by inducing “protective autophagy” [98].

*Maternally Expressed Gene 3 (MEG3)* encodes a tumor suppressor lncRNA that is expressed in many normal tissues [165]. Methylation of *MEG3* promoter and its marked downregulation have been reported in HCC cell lines and tissues [102,103]. *MEG3* expression negatively correlates with tumor size and TNM stage, thus acting as a potential prognostic biomarker [107]. The forced expression of *MEG3* in HCC cells significantly reduces both anchorage-dependent and -independent cell growth, and induces apoptosis [103], at least partially *via* the accumulation of p53 [107]. Indeed, it has been demonstrated that *MEG3* is able to interact with the p53 DNA binding domain [106], thus enhancing its stability and transcriptional activity.

*LincRNA-Ubiquitin-Fold Modifier Conjugating Enzyme 1 (lincRNA-UFC1)* is also upregulated in HCC tissues and its expression associates with tumor size, stage, and patient outcome. Its expression in HCC cells promotes cell proliferation and cell-cycle progression and inhibits apoptosis [166]. Levels of *lincRNA-UFC1* were described to correlate with those of ß-catenin in HCC tissues through a mechanism that involves the stabilization of the HuR protein (encoded by *ELAVL1*) by directly binding with the mRNA [166].

*Urothelial carcinoma-associated 1 (UCA1)* was reported to be markedly upregulated in HCC tissues and its expression in HCC is positively associated with tumor size, vascular invasion, TNM stage, metastasis, and postoperative survival [114,115]. Moreover, higher levels of *UCA1* were also detected in serum of HCC patients [167] and are associated with higher grade, larger tumor size, higher TNM stage, and vascular invasion, acting as an independent unfavorable prognostic factor for HCC [113]. Acting as a miRNA sponge, *UCA1* can either promote EMT [114] or activate the ERK signaling pathway in HCC [115]. Of note for hepatocarcinogenesis associated with HBV infection, *UCA1* was found to be frequently upregulated in HBx-positive tissues and was shown to be upregulated by HBx in hepatoma cells, thus promoting cell growth by facilitating G1/S transition through CDK2 [168].

*LncRNA-activated by TGF-β (lncRNA-ATB)* is significantly upregulated in HCC tissues and metastasis, and its expression is associated with poor prognosis [169,170]. At the molecular level, it has been shown that *lncRNA-ATB* can promote the invasion-metastasis cascade, either by inducing EMT through the upregulation of ZEB1 and ZEB2 or by binding *IL-11* mRNA and thus triggering STAT3 signaling [170]. These findings suggest that *lncRNA-ATB* predisposes HCC patients to metastasis and may potentially serve as a target for anti-metastatic therapies.

*High Expression In HCC (HEIH)* is another lncRNA whose high expression levels in HBV-related HCC were found to be significantly associated with recurrence and it was considered as an independent prognostic factor for survival. In patients with HCV-related HCC, *HEIH* expression in serum and exosomes is increased, but the ratio of *HEIH* expression in serum versus exosomes is decreased compared to patients with combined hepatocellular cholangiocarcinoma [81]. It was also described to play a key role in G0/G1 arrest and the same authors demonstrated that *HEIH* binds the enhancer of zeste homolog 2 (EZH2) factor inducing the repression of EZH2 target genes [80].

*PCNA Antisense RNA 1 (PCNA-AS1)* acts as a scaffold for mRNA molecules and was found significantly upregulated in HCC. Indeed, one of the roles of *PCNA-AS1* is the regulation of *PCNA* mRNA stability [171].

## 5. Putative Diagnostic and Prognostic lncRNAs in HCC

**Figure 3:**
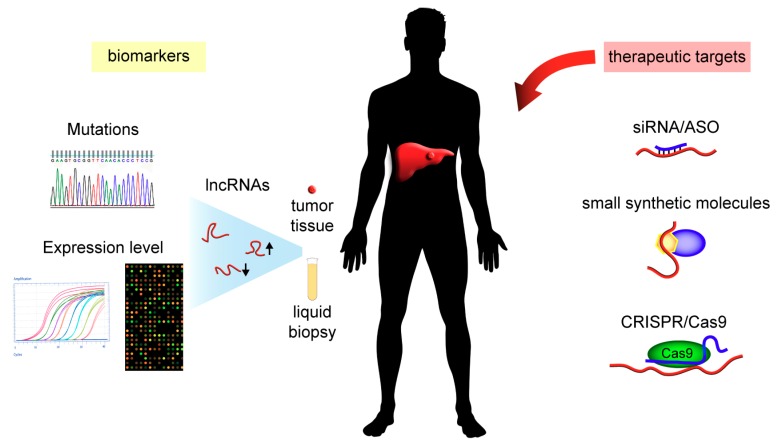
Diagnostic/prognostic and therapeutic potentials of lncRNAs. LncRNAs isolated from liquid biopsies or tissues could be analyzed at the sequence or expression levels and may serve as potential biomarkers for HCC diagnosis, prognosis and therapy response prediction. LncRNAs may also be targeted for therapeutic interventions by silencing their expression using canonical Argonaute2 mediated interference (siRNA) molecules or antisense oligonucleotides (ASO), by blocking the interactions with DNA, RNA, or proteins using small synthetic molecules or by CRISPR/Cas9 editing.

The identification of lncRNAs whose expression levels correlated with clinicopathological characteristics of patients led to many studies of their diagnostic and/or prognostic potential in HCC tissues and in liquid biopsies (Figure 3).

For instance, *UCA1* levels in HCC tissues [114,115] and in serum [113] were associated with high tumor grade, large tumor size, positive vascular invasion, and advanced TNM stage and may be an independent prognostic indicator [113]. Similarly, high levels of *MALAT1* were associated with reduced disease-free survival in patients after liver transplantation [100]. The association between *MALAT1* and prognosis appears to extend to plasma, with increased levels of *MALAT1* correlating with liver damage and predicting progression to HCC [172]. Interestingly, it has also been reported that germline variants of *MALAT1* and *HULC* may be associated with a decreased risk of HBV-associated HCC in the Chinese population [173].

Besides *UCA1*, *MALAT1*, and well-known lncRNAs, such as *HOTAIR* [82,84], *HULC* [92,93,174], a number of other lncRNAs whose expression was associated with disease stage and/or other clinicopathologic parameters have also been described. Recently, Yang et al., identified *HAND2-AS1* as a potential biomarker for HCC tumorigenesis and metastasis [75]. Similarly, high expression levels of *CASC15* [175], *CYTOR* (also known as *Linc00152*) [174], *HANR* [176], *ICR* (ICAM-1-Related ncRNA) [177], *linc-UFC1* [166], *lncRNA-ATB* [170], *lncSHRG* [178], *MVIH* [179], *PANDAR* [180], *PCAT14* [181], *SNHG6* [182], *SNHG20* [183], *TINCR* [184], *TMCC1-AS1* [72], *UBE2CP3* [185], *WRAP53* [116]], *ZEB1-AS1* [186], as well as the downregulation of *LOC728290* [187], *GAS5* [188], *DILC* [189], or *WT1-AS* [190], have been variably shown to be correlated with clinical severity, aggressive pathological features, metastasis, and/or poor outcome in HCC patients (Table S1). However, it should be noted that given that lncRNA is a fairly young research field, many of these associations have thus far been reported in single studies. Moreover, these studies differ substantially in their cohort sizes, etiologies and detection methods, and therefore the clinical utility for many of these lncRNAs remains to be validated and further tested in independent studies.

## 6. Therapeutic Potential of lncRNA in HCC

Tumor suppressor genes are notoriously difficult to target therapeutically, since a gain of function is difficult to achieve using current generations of therapeutic options [191]. In this context, the ability for lncRNAs to variably up- or down-regulate coding genes makes them attractive therapeutic targets. In particular, one possibility is the modulation of cis-acting lncRNAs, which may result in specific, endogenous alteration of the expression level of their target genes [192]. Several approaches have been proposed to target the various aspects of lncRNA mechanisms of action (Table 2). It should, however, be noted that lncRNAs as therapeutics is currently largely speculative based on the biological functions of lncRNAs and data observed in in vitro/in vivo studies. The development of therapeutic agents against lncRNAs is still far from clinical application.

The first proposed approaches directly target lncRNAs to induce their degradation or destabilization. These methods include RNA interference mediated gene silencing and antisense oligonucleotides (ASO). For example, the delivery of siRNA molecules using ultrasound-targeted microbubble destruction was used to silence *lncRNA-ATB*, suppressing HCC migration and invasion in vitro [195]. Some in vivo evidence of successful inhibition of *MALAT1* and metastasis by injecting ASO into subcutaneous tumors of nude mice has been reported for lung cancer cells [196].

An alternative therapeutic approach could be to block the interactions of lncRNA with DNA, RNA, or proteins using antagonistic sequences or small synthetic molecules that cover the lncRNA binding sites [198]. In addition, gene therapy represents an emerging and very promising strategy. Indeed, it was recently shown that the CRISPR/Cas9 technology could be successfully used to target an enhancer and exonic fragment of *MALAT1* in human cells [197].

In view of their miRNA binding capacity, lncRNAs can be used not only as targets but also to target miRNAs involved in HCC. For example, the miRNAs *miR-21*, *miR-153*, *miR-216a*, *miR-217*, *miR-494* and *miR-10a-5p* have been shown to be upregulated in sorafenib-resistant cells and to participate in the mechanisms that are underlying sorafenib resistance [193]. The simultaneous targeting of these miRNAs using an artificial lncRNA expressed by an adenoviral vector (Ad5-AlncRNA) inhibits proliferation and induces apoptosis of sorafenib-resistant cells and enhances the effects of sorafenib in vitro and in vivo [193]. This may represent a potential strategy to overcome sorafenib resistance in the treatment of HCC.

## 7. Conclusions

Whole-transcriptome analyses are beginning to provide important insights into the biological and clinical relevance of lncRNAs in cancer. When compared to protein-coding genes, our knowledge in lncRNAs is in its infancy and many, many more studies are required to define which lncRNAs are genuinely critical in hepatocarcinogenesis. For HCC, the lack of molecular targets may benefit from exploiting lncRNAs as therapeutic targets. Future development in this area will be particularly exciting to increase the number of treatment options.

## Figures and Tables

**Table 1:** LncRNAs dysregulated in HCC.

lncRNA	Class	Expression in HCC	Effect on HCC	Molecular Mechanism	Reference
*H19*	intergenic	downregulated/upregulated	Inhibits migration and invasion; associated with HCC aggressiveness and poor outcome; promotes cell growth	Recruits HnRNP U/PCAF/RNAPolII complex to activate *miR-200* family through histone acetylation;is involved in the miR675/AKT/GSK-3beta/Cdc25A signaling pathway; interacts with EZH2 and represses E-caderin expression	[77,78,79]
*HEIH*	intergenic	upregulated	Promotes tumor progression and inhibits G0/G1 cell cycle arrest	Interacts with EZH2 and represses target genes	[80,81]
*HOTAIR*	intergenic	upregulated	Promotes cell proliferation, viability, invasion, migration and metastasis; suppresses apoptosis	Regulates miR-331-3p/*HER2*, miR-1/FOXC1, miR-218/BMI1/Ink4a/ARF, DDX5/PRC2, STAT3, GLUT1/mTOR signaling pathways	[82,83,84,85,86,87,88]
*HOTTIP*	intergenic	upregulated	Promotes cell proliferation, migration, tumorigenesis and metastasis	Interacts with WDR5/MLL promoting H3K4me3	[89,90]
*HULC*	intergenic	upregulated	Promotes cell growth, proliferation, EMT, migration, tumor progression, metastasis, angiogenesis; modulates lipid metabolism	Regulates several signaling pathways including miR-9/PPARA/ACSL1, miR-200a-3p/ZEB1, miR-107/E2F1/SPHK1, miR-488/ADAM, mR186/HMGA2; regulates the ubiquitin-mediated degradation of Sirt1	[91,92,93,94,95,96,97,98]
*MALAT1*	antisense	upregulated	Promotes cell invasion, migration, growth, motility and metastasis	Sponges miR-125b, miR-146b-5p, miR-204, miR-143-3p, miR-195; regulates p53/DBC1 signaling pathway	[63,99,100,101]
*MEG3*	intergenic	downregulated	Promotes proliferation and apoptosis	Regulates p53 transcription	[102,103,104,105,106,107]
*NEAT1*	intergenic	upregulated	Promotes tumorigenesis, EMT, cell proliferation, migration and metastasis	Regulates the miR-129-5p/VCP/IkB axis; sponges *miR-613*; regulates STAT3 expression through *miR-485*; regulates hnRNP A2 expression by sequestrating U2AF65; is involved in paraspeckle formation	[108,109,110,111,112]
*UCA1*	intergenic	upregulated	Promotes EMT; is associated with tumor size, vascular invasion, TNM stage, metastasis and postoperative survival	Activates ERK signaling pathway; regulates SNAIL2 expression	[113,114,115,116]

EMT: epithelial to mesenchymal transition; TNM: tumor/node/metastasis.

**Table 2:** Therapeutic use of lncRNAs in HCC.

lncRNA	Molecular Strategy	Reference
*Ad5-AlncRNA*	Overexpressed to target miRNAs	[193]
*DANCR*	Silenced by shRNA	[194]
*lncRNA-ATB*	Silenced by siRNA molecules	[195]
*MALAT1*	Silenced by antisense oligonucleotides (ASO)	[196]
*MALAT1*	Silenced by CRISPR/Cas9	[197]

## Supplementary Materials

Supplementary materials can be found at http://www.mdpi.com/1422-0067/19/3/682/s1.

## Abbreviations

LncRNA	Long non-Coding RNA
HCC	Hepatocellular Carcinoma
HBV	Hepatitis B Virus
HCV	Hepatitis C Virus
TCGA	The Cancer Genome Atlas
Sno-RNA	Small nuclear RNA
CircRNA	Circular Intronic Long non-Coding RNA
SnolncRNA	Small Nuclear Long non-Coding RNA
NAT	Natural Antisense Transcript
LincRNA	Long intergenic RNA
PancRNA	Promoter Associated non-Coding RNA
CeRNA	Competing Endogenous RNA
EMT	Epithelial to Mesenchymal Transition
TNM	Tumor Node Metastasis
eRNA	Enhancer RNA
siRNA	Small interference RNA
MET	Mesenchimal to Epithelial Transition
ASO	Antisense Oligonucleotides
