# Supplementary material for: The Role of Long Non-Coding RNAs in Hepatocarcinogenesis
Source: Int J Mol Sci. 2018 Feb 28;19(3):682. doi: 10.3390/ijms19030682 (PMC5877543; doi:10.3390/ijms19030682)
Supplement: Supplementary file 1 [file ijms-19-00682-s001.pdf]

Supplementary Materials

Table S1. Comprehensive list of dysregulated lncRNAs in hepatocellular carcinomas.

lncRNA	Class	Dysregulation in HCC	Effect on HCC	Function	Reference
AB019562	antisense	upregulated	Promotes cell proliferation and metastasis, suppresses of apoptosis	-	[1]
AB209630	antisense	downregulated	Suppresses cell proliferation and metastasis	-	[2]
AF070632	intergenic	downregulated	Potential biomarker	-	[3]
AF113014	antisense	downregulated	Inhibits cell proliferation and tumor growth	miRNA sponge	[4]
ANRIL	antisense	upregulated	Promotes cell proliferation, metastasis, and invasion, suppresses apoptosis; associated with poor prognosis	epigenetic modification	[3,5–7]
AOC4P	intergenic	downregulated	Inhibits cell proliferation, migration, and invasion by regulating EMT	-	[8]
BANCR	intergenic	upregulated	Promotes cell proliferation, migration and invasion; suppresses apoptosis via inhibition of the activity of MEK	-	[9]
CARLo-5	intergenic	upregulated	Promotes cell proliferation, migration, invasion, metastasis	-	[10]
CASC15	intergenic	upregulated	Promotes cell proliferation, migration, invasionand tumor growth; inhibit apoptosis	-	[11]
CASC2	intergenic	downregulated	Inhibits cell migration, invasion, EMT and tumor growth; induces apoptosis	miRNA sponge	[12–14]
DANCR	intergenic	upregulated	Promotes stemness, cell vitality, tumor shrinkage	mRNA binding	[15]
DBH-AS1	antisense	upregulated	Promotes cell proliferation, migration; suppresses apoptosis	-	[16]
DILC	non-coding transcript variant	downregulated	Enhances liver cancer stem cells expansion and promotes HCC initiation and progression	chromatin remodeling	[17]
DREH	intergenic	downregulated	Inhibits cell proliferation, growth and metastasis	-	[18,19]
FTX	intergenic	downregulated	Inhibits cell proliferation and metastasis	miRNA sponge, protein binding	[20–22]
GAS5	intergenic	downregulated	Suppresses cell proliferation, migration, invasion and apoptosis	miRNA sponge	[23–26]
GPC3-AS1	antisense	upregulated	Promotes cell proliferation and migration in vitro and xenograft tumor growth in vivo	epigenetic modification	[27,28]

HAND2-AS1	intergenic	upregulated	Promotes metastasis	-	[29]
HANR	intergenic	upregulated	Promotes cell proliferation, tumor growth, inhibit apoptosis and chemosensitivity to doxorubicin	protein binding	[30]
HNF1A-AS1	antisense	upregulated	Regulates tumor growth and apoptosis promotes cell proliferation and S-phase progression	miRNA sponge, protein binding	[31,32]
HOXD-AS1	intergenic	upregulated	Promotes cancer metastasis and inhibits apoptosis	miRNA sponge	[33,34]
HOXD-AS2	intergenic	upregulated	Promotes proliferation and invasion and inhibits apoptosis.	-	[35]
ICR	antisense	upregulated	Promotes portal vein tumor thrombus (PVTT) through stabilizing ICAM-1	mRNA binding	[36]
Igf2as	antisense	upregulated	Promotes cell proliferation and inhibit apoptosis	-	[37]
INTS6P1	intergenic	downregulated	Inhibits cell growth and migration	miRNA sponge	[38,39]
linc00152	intergenic	upregulated	Diagnostic candidate	chromatin remodeling	[40]
linc00176	intergenic	upregulated	Regulates cell cycle and cell survival	miRNA sponge	[41]
linc00441	bidirectional	upregulated	Promotes the proliferation, inhibits apoptosis and cell cycle arrest	epigenetic modification	[42]
linc00462	intergenic	upregulated	Promotes cancer progression	-	[43]
linc00657	intergenic	upregulated	Promotes proliferation and invasion	miRNA sponge	[44]
linc00974	intergenic	upregulated	Promotes cell proliferation and invasion, inhibit apoptosis	miRNA sponge	[45]
linc01225	intergenic	upregulated	Promotes proliferation, invasion, inhibits apoptosis	protein binding	[46]
linc01419	intergenic	upregulated	Predicted to regulate cell cycle	-	[47]
lincRNA-p21	intergenic	downregulated	Inhibits migration and invasion	miRNA sponge	[48,49]
lincRNA-UFC1	intergenic	upregulated	Promotes cell proliferation, cell migration and invasion and inhibits G0/G1 cell cycle arrest	protein binding	[50]
linc-ROR	intergenic	upregulated	Confers chemoresistance	miRNA sponge	[51]
lnc-beta-Catm	intergenic	upregulated	Promotes self-renewal of liver cscs and tumor propagation	protein binding	[52]
LncBRM	intergenic	upregulated	Promotes self-renewal maintenance of liver cscs and tumour initiation	chromatin remodeling	[53]
lncRNA00673	intergenic	upregulated	Promotes cell proliferation	-	[54]

LncRNA-AFAP1-AS1	intergenic	upregulated	Promotes cell proliferation, clonal growth, cell migration and invasion; inhibits apoptosis	-	[55,56]
LncRNA-ATB	intergenic	upregulated	Promotes EMT, cell invasion and metastasis	miRNA sponge, mRNA binding	[57–59]
lncRNA-CPS1-IT1	intronic	downregulated	Inhibits cell proliferation, migration, invasion, EMT and metastasis	protein binding	[60,61]
lncRNA-GIHCG	undefined	upregulated	Promotes cell proliferation, migration, invasion and metastasis	epigenetic modification	[62]
lncRNA-hPVT1	intergenic	upregulated	Promotes proliferation and stem cell-like property	protein binding	[63,64]
lncRNA-MUF	intronic	upregulated	Promotes EMT *via* WNT/beta-catenin signaling;	miRNA sponge, protein binding	[65]
lncSHRG	intergenic	upregulated	Promotes tumor cell proliferation	protein binding	[66]
LncSox4	non-coding transcript variant	upregulated	Promotes tumor progression	protein binding	[67]
LncTCF7	intergenic	upregulated	Promotes tumor growth and propagation, and EMT	chromatin remodeling	[68,69]
LOC728290	intergenic	downregulated	Potential tumor biomarker	-	[70]
LOC90784	intergenic	upregulated	Promotes cell proliferation, invasion and migration		[71]
MIAT	intergenic	upregulated	Promotes cell growth, motility, invasion, migration and metastasis	miRNA sponge	[72]
MVIH	intergenic	upregulated	Promotes tumor growth and intrahepatic metastasis by activating angiogenesis; promotes cell proliferation and invasion	miRNA sponge, protein binding	[74]
OCT4-pg4	pseudogene	upregulated	Promotes growth and tumorigenicity	miRNA sponge	[75]
PANDAR	anti-sense	upregulated	Promotes cell proliferation, colony formation and cycle progression	-	[76]
PCAT-1	intergenic	upregulated	Promotes cell proliferation, migration, apoptosis, invasion and metastasis	miRNA sponge	[77–80]
PCAT-14	intergenic	upregulated	Promotes proliferation, invasion, cell cycle arrest	epigenetic modification	[81]
PCNA-AS1	antisense	upregulated	Promotes tumor growth	mRNA binding	[82,83]
PDIA3P1	antisense	upregulated	Promotes cell proliferation, migration, and invasion; suppresses apoptosis	-	[84]
PRAL	intergenic	downregulated	Inhibits cell growth	protein binding	[85]

PTENP1	intergenic	downregulated	Inhibits cell proliferation, invasion, metastasis and angiogenesis; promotes apoptosis	miRNA sponge	[86,87]
PVT1	intergenic	upregulated	Promotes cell proliferation, stem cell-like properties of HCC cells, tumor progression	miRNA sponge, protein binding	[88–92]
SNHG20	sense-overlapping	upregulated	Promotes cells proliferation, migration, and invasion	chromatin remodeling	[93,94]
SNHG6	antisense	upregulated	Promotes cellular proliferation, migration, and invasion	miRNA sponge, protein binding	[95]
SNHG6-003	antisense	upregulated	Promotes cell proliferation, induces drug resistance and promotes apoptosis	miRNA sponge	[96]
SNHG7	intergenic	upregulated	Promotes cell proliferation, stem cell-like properties of HCC cells, tumor progression	-	[88]
SPRY4-IT1	intronic	upregulated	Promotes cell proliferation, colony formation, cell invasion, migration and EMT; inhibit cell cycle arrest at G0/G1 and apoptosis	epigenetic modification, protein binding	[97–99]
TINCR	intronic	upregulated	Promotes differentiation, invasion, and metastasis	miRNA sponge	[100]
TUC338	sense	upregulated	Promotes cell growth, proliferation and cell cycle progression	chromatin remodeling	[101,102]
TUG1	intergenic	upregulated	Promotes cell proliferation, colony formation, tumorigenicity; suppresses apoptosis	miRNA sponge, epigenetic modification	[103–105]
UBE2CP3	intergenic	upregulated	Induces EMT	-	[106]
UCA1	intergenic	upregulated	Promotes cell growth, proliferation, invasion and metastasis	miRNA sponge	[107–110]
WRAP53	intergenic	upregulated	Promotes cell proliferation and invasion	-	[111]
WT1-AS	bidirectional	downregulated	Promotes apoptosis by binding to the promoter region of WT1	chromatin remodeling	[112]
ZEB1-AS1	antisense	upregulated	Promotes tumor growth, EMT and metastasis	chromatin remodeling	[113]
ZFAS1	antisense	upregulated	Promotes cell invasion and metastasis	miRNA sponge	[114]

Reference

1. Wu, F.; Li, J.; Du, X.; Zhang, W.; Lei, P.; Zhang, Q. Long noncoding RNA AB019562 promotes cell proliferation and metastasis in human hepatocellular carcinoma. *Mol. Med. Rep.* **2017**, *16*, 69–74, doi:10.3892/mmr.2017.6612.
2. Li, T.; Liu, Y.; Sun, Y. Long non-coding RNA ab209630 suppresses cell proliferation and metastasis in human hepatocellular carcinoma. *Exp. Ther. Med.* **2017**, *14*, 3419–3424, doi:10.3892/etm.2017.4927.

3. Zhang, H.; Zhu, C.; Zhao, Y.; Li, M.; Wu, L.; Yang, X.; Wan, X.; Wang, A.; Zhang, M.Q.; Sang, X.; et al. Long non-coding RNA expression profiles of hepatitis C virus-related dysplasia and hepatocellular carcinoma. *Oncotarget* **2015**, *6*, 43770–43778, doi:10.18632/oncotarget.6087.
4. Zeng, T.; Wang, D.; Chen, J.; Tian, Y.; Cai, X.; Peng, H.; Zhu, L.; Huang, A.; Tang, H. LncRNA-Af113014 promotes the expression of EGR2 by interaction with miR-20a to inhibit proliferation of hepatocellular carcinoma cells. *PLoS ONE* **2017**, *12*, e0177843, doi:10.1371/journal.pone.0177843.
5. Ma, J.; Li, T.; Han, X.; Yuan, H. Knockdown of lncrna anril suppresses cell proliferation, metastasis, and invasion via regulating miR-122-5p expression in hepatocellular carcinoma. *J. Cancer Res. Clin. Oncol.* **2017**, doi:10.1007/s00432-017-2543-y.
6. Huang, M.D.; Chen, W.M.; Qi, F.Z.; Xia, R.; Sun, M.; Xu, T.P.; Yin, L.; Zhang, E.B.; De, W.; Shu, Y.Q. Long non-coding RNA anril is upregulated in hepatocellular carcinoma and regulates cell proliferation by epigenetic silencing of KLF2. *J. Hematol. Oncol.* **2015**, *8*, 57, doi:10.1186/s13045-015-0153-1.
7. Hua, L.; Wang, C.Y.; Yao, K.H.; Chen, J.T.; Zhang, J.J.; Ma, W.L. High expression of long non-coding RNA anril is associated with poor prognosis in hepatocellular carcinoma. *Int. J. Clin. Exp. Pathol.* **2015**, *8*, 3076–3082.
8. Wang, T.H.; Lin, Y.S.; Chen, Y.; Yeh, C.T.; Huang, Y.L.; Hsieh, T.H.; Shieh, T.M.; Hsueh, C.; Chen, T.C. Long non-coding RNA AOC4P suppresses hepatocellular carcinoma metastasis by enhancing vimentin degradation and inhibiting epithelial-mesenchymal transition. *Oncotarget* **2015**, *6*, 23342–23357, doi:10.18632/oncotarget.4344.
9. Li, J.; Wang, J.; Zhou, W.; Zhang, S.; Le, Y.; He, R. Downregulation of braf-activated non-coding RNA suppresses the proliferation, migration and invasion, and induces apoptosis of hepatocellular carcinoma cells. *Oncol. Lett.* **2017**, *14*, 4751–4757, doi:10.3892/ol.2017.6770.
10. Wang, F.; Xie, C.; Zhao, W.; Deng, Z.; Yang, H.; Fang, Q. Long non-coding RNA carlo-5 expression is associated with disease progression and predicts outcome in hepatocellular carcinoma patients. *Clin. Exp. Med.* **2017**, *17*, 33–43, doi:10.1007/s10238-015-0395-9.
11. He, T.; Zhang, L.; Kong, Y.; Huang, Y.; Zhang, Y.; Zhou, D.; Zhou, X.; Yan, Y.; Zhang, L.; Lu, S.; et al. Long non-coding RNA CASC15 is upregulated in hepatocellular carcinoma and facilitates hepatocarcinogenesis. *Int. J. Oncol.* **2017**, *51*, 1722–1730, doi:10.3892/ijo.2017.4175.
12. Wang, Y.; Liu, Z.; Yao, B.; Li, Q.; Wang, L.; Wang, C.; Dou, C.; Xu, M.; Liu, Q.; Tu, K. Long non-coding RNA CASC2 suppresses epithelial-mesenchymal transition of hepatocellular carcinoma cells through CASC2/miR-367/FBXW7 axis. *Mol. Cancer* **2017**, *16*, 123, doi:10.1186/s12943-017-0702-z.
13. Zeng, F.; Le, Y.G.; Fan, J.C.; Xin, L. Lncrna CASC2 inhibited the viability and induced the apoptosis of hepatocellular carcinoma cells through regulating miR-24-3p. *J. Cell. Biochem.* **2017**, doi:10.1002/jcb.26479.
14. Zhao, L.; Zhang, Y.; Zhang, Y. Long noncoding RNA CASC2 regulates hepatocellular carcinoma cell oncogenesis through miR-362-5p/Nf-kappaB axis. *J. Cell. Physiol.* **2018**, doi:10.1002/jcp.26446.
15. Yuan, S.X.; Wang, J.; Yang, F.; Tao, Q.F.; Zhang, J.; Wang, L.L.; Yang, Y.; Liu, H.; Wang, Z.G.; Xu, Q.G.; et al. Long noncoding RNA dancr increases stemness features of hepatocellular carcinoma by derepression of CTNNB1. *Hepatology* **2016**, *63*, 499–511, doi:10.1002/hep.27893.
16. Huang, J.L.; Ren, T.Y.; Cao, S.W.; Zheng, S.H.; Hu, X.M.; Hu, Y.W.; Lin, L.; Chen, J.; Zheng, L.; Wang, Q. Hbx-related long non-coding RNA DBH-AS1 promotes cell proliferation and survival by activating mapk signaling in hepatocellular carcinoma. *Oncotarget* **2015**, *6*, 33791–33804, doi:10.18632/oncotarget.5667.
17. Wang, X.; Sun, W.; Shen, W.; Xia, M.; Chen, C.; Xiang, D.; Ning, B.; Cui, X.; Li, H.; Li, X.; et al. Long non-coding RNA dilc regulates liver cancer stem cells via IL-6/STAT3 axis. *J. Hepatol.* **2016**, *64*, 1283–1294, doi:10.1016/j.jhep.2016.01.019.
18. Huang, J.F.; Guo, Y.J.; Zhao, C.X.; Yuan, S.X.; Wang, Y.; Tang, G.N.; Zhou, W.P.; Sun, S.H. Hepatitis b virus x protein (HBX)-related long noncoding RNA (lncRNA) down-regulated expression by HBX (DREH) inhibits hepatocellular carcinoma metastasis by targeting the intermediate filament protein vimentin. *Hepatology* **2013**, *57*, 1882–1892, doi:10.1002/hep.26195.
19. Lv, D.; Wang, Y.; Zhang, Y.; Cui, P.; Xu, Y. Downregulated long non-coding RNA dreh promotes cell proliferation in hepatitis B virus-associated hepatocellular carcinoma. *Oncol. Lett.* **2017**, *14*, 2025–2032, doi:10.3892/ol.2017.6436.
20. Liu, F.; Yuan, J.H.; Huang, J.F.; Yang, F.; Wang, T.T.; Ma, J.Z.; Zhang, L.; Zhou, C.C.; Wang, F.; Yu, J.; et al. Long noncoding RNA FTX inhibits hepatocellular carcinoma proliferation and metastasis by binding MCM2 and miR-374a. *Oncogene* **2016**, *35*, 5422–5434, doi:10.1038/onc.2016.80.
21. Liu, Z.; Dou, C.; Yao, B.; Xu, M.; Ding, L.; Wang, Y.; Jia, Y.; Li, Q.; Zhang, H.; Tu, K.; et al. Ftx non coding RNA-derived miR-545 promotes cell proliferation by targeting rig-I in hepatocellular carcinoma. *Oncotarget* **2016**, *7*, 25350–25365, doi:10.18632/oncotarget.8129.

22. Zhao, Q.; Li, T.; Qi, J.; Liu, J.; Qin, C. The miR-545/374a cluster encoded in the FTX lncRNA is overexpressed in HBV-related hepatocellular carcinoma and promotes tumorigenesis and tumor progression. *PLoS ONE* **2014**, *9*, e109782, doi:10.1371/journal.pone.0109782.
23. Tu, Z.Q.; Li, R.J.; Mei, J.Z.; Li, X.H. Down-regulation of long non-coding RNA GAS5 is associated with the prognosis of hepatocellular carcinoma. *Int. J. Clin. Exp. Pathol.* **2014**, *7*, 4303–4309.
24. Chang, L.; Li, C.; Lan, T.; Wu, L.; Yuan, Y.; Liu, Q.; Liu, Z. Decreased expression of long non-coding RNA GAS5 indicates a poor prognosis and promotes cell proliferation and invasion in hepatocellular carcinoma by regulating vimentin. *Mol. Med. Rep.* **2016**, *13*, 1541–1550, doi:10.3892/mmr.2015.4716.
25. Hu, L.; Ye, H.; Huang, G.; Luo, F.; Liu, Y.; Liu, Y.; Yang, X.; Shen, J.; Liu, Q.; Zhang, J. Long noncoding RNA GAS5 suppresses the migration and invasion of hepatocellular carcinoma cells via miR-21. *Tumour Biol.* **2016**, *37*, 2691–2702, doi:10.1007/s13277-015-4111-x.
26. Tao, R.; Hu, S.; Wang, S.; Zhou, X.; Zhang, Q.; Wang, C.; Zhao, X.; Zhou, W.; Zhang, S.; Li, C.; et al. Association between indel polymorphism in the promoter region of lncRNA GAS5 and the risk of hepatocellular carcinoma. *Carcinogenesis* **2015**, *36*, 1136–1143, doi:10.1093/carcin/bgv099.
27. Lu, J.; Xie, F.; Geng, L.; Shen, W.; Sui, C.; Yang, J. Investigation of serum lncRNA-uc003WBD and LncRNA-AF085935 expression profile in patients with hepatocellular carcinoma and HBV. *Tumour Biol* **2015**, *36*, 3231–3236, doi:10.1007/s13277-014-2951-4.
28. Zhu, X.T.; Yuan, J.H.; Zhu, T.T.; Li, Y.Y.; Cheng, X.Y. Long noncoding RNA glypican 3 (GPC3) antisense transcript 1 promotes hepatocellular carcinoma progression via epigenetically activating GPC3. *FEBS J.* **2016**, *283*, 3739–3754, doi:10.1111/febs.13839.
29. Yang, Y.; Chen, L.; Gu, J.; Zhang, H.; Yuan, J.; Lian, Q.; Lv, G.; Wang, S.; Wu, Y.; Yang, Y.T.; et al. Recurrently deregulated lncrnas in hepatocellular carcinoma. *Nat. Commun.* **2017**, *8*, 14421, doi:10.1038/ncomms14421.
30. Xiao, J.; Lv, Y.; Jin, F.; Liu, Y.; Ma, Y.; Xiong, Y.; Liu, L.; Zhang, S.; Sun, Y.; Tipoe, G.L.; et al. Lncrna hanr promotes tumorigenesis and increase of chemoresistance in hepatocellular carcinoma. *Cell. Physiol. Biochem.* **2017**, *43*, 1926–1938, doi:10.1159/000484116.
31. Liu, Z.; Wei, X.; Zhang, A.; Li, C.; Bai, J.; Dong, J. Long non-coding RNA HNF1A-AS1 functioned as an oncogene and autophagy promoter in hepatocellular carcinoma through sponging HSA-miR-30b-5p. *Biochem. Biophys. Res. Commun.* **2016**, *473*, 1268–1275, doi:10.1016/j.bbrc.2016.04.054.
32. Wang, C.; Mou, L.; Chai, H.X.; Wang, F.; Yin, Y.Z.; Zhang, X.Y. Long non-coding RNA HNF1A-AS1 promotes hepatocellular carcinoma cell proliferation by repressing NKD1 and p21 expression. *Biomed. Pharmacother.* **2017**, *89*, 926–932, doi:10.1016/j.biopha.2017.01.031.
33. Wang, H.; Huo, X.; Yang, X.R.; He, J.; Cheng, L.; Wang, N.; Deng, X.; Jin, H.; Wang, N.; Wang, C.; et al. STAT3-mediated upregulation of lncrna HOXD-AS1 as a cerna facilitates liver cancer metastasis by regulating SOX4. *Mol. Cancer* **2017**, *16*, 136, doi:10.1186/s12943-017-0680-1.
34. Lu, S.; Zhou, J.; Sun, Y.; Li, N.; Miao, M.; Jiao, B.; Chen, H. The noncoding RNA HOXD-AS1 is a critical regulator of the metastasis and apoptosis phenotype in human hepatocellular carcinoma. *Mol. Cancer* **2017**, *16*, 125, doi:10.1186/s12943-017-0676-x.
35. Wang, F.; Yang, H.; Deng, Z.; Su, Y.; Fang, Q.; Yin, Z. HOX antisense lincRNA *HOXA*-AS2 promotes tumorigenesis of hepatocellular carcinoma. *Cell. Physiol. Biochem.* **2016**, *40*, 287–296, doi:10.1159/000452545.
36. Guo, W.; Liu, S.; Cheng, Y.; Lu, L.; Shi, J.; Xu, G.; Li, N.; Cheng, K.; Wu, M.; Cheng, S.; et al. ICAM-1-related noncoding RNA in cancer stem cells maintains ICAM-1 expression in hepatocellular carcinoma. *Clin. Cancer Res.* **2016**, *22*, 2041–2050, doi:10.1158/1078-0432.CCR-14-3106.
37. Bao, H.; Guo, C.G.; Qiu, P.C.; Zhang, X.L.; Dong, Q.; Wang, Y.K. Long non-coding RNA IGF2AS controls hepatocellular carcinoma progression through the ERK/MAPK signaling pathway. *Oncol. Lett.* **2017**, *14*, 2831–2837, doi:10.3892/ol.2017.6492.
38. Lui, K.Y.; Peng, H.R.; Lin, J.R.; Qiu, C.H.; Chen, H.A.; Fu, R.D.; Cai, C.J.; Lu, M.Q. Pseudogene integrator complex subunit 6 pseudogene 1 (INTS6P1) as a novel plasma-based biomarker for hepatocellular carcinoma screening. *Tumour Biol.* **2016**, *37*, 1253–1260, doi:10.1007/s13277-015-3899-8.
39. Peng, H.; Ishida, M.; Li, L.; Saito, A.; Kamiya, A.; Hamilton, J.P.; Fu, R.; Olaru, A.V.; An, F.; Popescu, I.; et al. Pseudogene INTS6P1 regulates its cognate gene INTS6 through competitive binding of miR-17-5p in hepatocellular carcinoma. *Oncotarget* **2015**, *6*, 5666–5677, doi:10.18632/oncotarget.3290.
40. Li, J.; Wang, X.; Tang, J.; Jiang, R.; Zhang, W.; Ji, J.; Sun, B. Hulc and *Linc00152* act as novel biomarkers in predicting diagnosis of hepatocellular carcinoma. *Cell. Physiol. Biochem.* **2015**, *37*, 687–696, doi:10.1159/000430387.

41. Tran, D.D.H.; Kessler, C.; Niehus, S.E.; Mahnkopf, M.; Koch, A.; Tamura, T. Myc target gene, long intergenic noncoding RNA, *Linc00176* in hepatocellular carcinoma regulates cell cycle and cell survival by titrating tumor suppressor micrornas. *Oncogene* **2017**, doi:10.1038/onc.2017.312.
42. Tang, J.; Xie, Y.; Xu, X.; Yin, Y.; Jiang, R.; Deng, L.; Tan, Z.; Gangarapu, V.; Tang, J.; Sun, B. Bidirectional transcription of linc00441 and RB1 via H3K27 modification-dependent way promotes hepatocellular carcinoma. *Cell Death Dis.* **2017**, *8*, e2675, doi:10.1038/cddis.2017.81.
43. Gong, J.; Qi, X.; Zhang, Y.; Yu, Y.; Lin, X.; Li, H.; Hu, Y. Long noncoding RNA linc00462 promotes hepatocellular carcinoma progression. *Biomed. Pharmacother.* **2017**, *93*, 40–47, doi:10.1016/j.biopha.2017.06.004.
44. Hu, B.; Cai, H.; Zheng, R.; Yang, S.; Zhou, Z.; Tu, J. Long non-coding RNA 657 suppresses hepatocellular carcinoma cell growth by acting as a molecular sponge of miR-106a-5p to regulate pten expression. *Int. J. Biochem. Cell Biol.* **2017**, *92*, 34–42, doi:10.1016/j.biocel.2017.09.008.
45. Tang, J.; Zhuo, H.; Zhang, X.; Jiang, R.; Ji, J.; Deng, L.; Qian, X.; Zhang, F.; Sun, B. A novel biomarker linc00974 interacting with KRT19 promotes proliferation and metastasis in hepatocellular carcinoma. *Cell Death Dis.* **2014**, *5*, e1549, doi:10.1038/cddis.2014.518.
46. Wang, X.; Zhang, W.; Tang, J.; Huang, R.; Li, J.; Xu, D.; Xie, Y.; Jiang, R.; Deng, L.; Zhang, X.; et al. *Linc01225* promotes occurrence and metastasis of hepatocellular carcinoma in an epidermal growth factor receptor-dependent pathway. *Cell Death Dis.* **2016**, *7*, e2130, doi:10.1038/cddis.2016.26.
47. Zhang, H.; Zhu, C.; Zhao, Y.; Li, M.; Wu, L.; Yang, X.; Wan, X.; Wang, A.; Zhang, M.Q.; Sang, X.; et al. Long non-coding RNA expression profiles of hepatitis C virus-related dysplasia and hepatocellular carcinoma. *Oncotarget* **2015**, *6*, 43770–43778, doi:10.18632/oncotarget.6087.
48. Ding, G.; Peng, Z.; Shang, J.; Kang, Y.; Ning, H.; Mao, C. LincRNA-p21 inhibits invasion and metastasis of hepatocellular carcinoma through miR-9/e-cadherin cascade signaling pathway molecular mechanism. *OncoTargets Ther.* **2017**, *10*, 3241–3247, doi:10.2147/OTT.S134910.
49. Jia, M.; Jiang, L.; Wang, Y.D.; Huang, J.Z.; Yu, M.; Xue, H.Z. LincRNA-p21 inhibits invasion and metastasis of hepatocellular carcinoma through notch signaling-induced epithelial-mesenchymal transition. *Hepatol. Res.* **2016**, *46*, 1137–1144, doi:10.1111/hepr.12659.
50. Cao, C.; Sun, J.; Zhang, D.; Guo, X.; Xie, L.; Li, X.; Wu, D.; Liu, L. The long intergenic noncoding RNA UFC1, a target of microrna 34a, interacts with the mrna stabilizing protein hur to increase levels of beta-catenin in hcc cells. *Gastroenterology* **2015**, *148*, 415–426 e418, doi:10.1053/j.gastro.2014, doi:10.012.
51. Takahashi, K.; Yan, I.K.; Kogure, T.; Haga, H.; Patel, T. Extracellular vesicle-mediated transfer of long non-coding RNA ror modulates chemosensitivity in human hepatocellular cancer. *FEBS Open Bio* **2014**, *4*, 458–467, doi:10.1016/j.fob.2014.04.007.
52. Zhu, P.; Wang, Y.; Huang, G.; Ye, B.; Liu, B.; Wu, J.; Du, Y.; He, L.; Fan, Z. Lnc-beta-catm elicits EZH2-dependent beta-catenin stabilization and sustains liver csc self-renewal. *Nat. Struct. Mol. Biol.* **2016**, *23*, 631–639, doi:10.1038/nsmb.3235.
53. Zhu, P.; Wang, Y.; Wu, J.; Huang, G.; Liu, B.; Ye, B.; Du, Y.; Gao, G.; Tian, Y.; He, L.; et al. Lncbrm initiates YAP1 signalling activation to drive self-renewal of liver cancer stem cells. *Nat. Commun.* **2016**, *7*, 13608, doi:10.1038/ncomms13608.
54. Chen, H.; Liu, J.Z.; Hu, G.J.; Shi, L.L.; Lan, T. Promotion of proliferation and metastasis of hepatocellular carcinoma by lncRNA00673 based on the targeted-regulation of notch signaling pathway. *Eur. Rev. Med. Pharmacol. Sci.* **2017**, *21*, 3412–3420.
55. Lu, X.; Zhou, C.; Li, R.; Liang, Z.; Zhai, W.; Zhao, L.; Zhang, S. Critical role for the long non-coding RNA AFAP1-AS1 in the proliferation and metastasis of hepatocellular carcinoma. *Tumour Biol.* **2016**, *37*, 9699–9707, doi:10.1007/s13277-016-4858-8.
56. Zhang, J.Y.; Weng, M.Z.; Song, F.B.; Xu, Y.G.; Liu, Q.; Wu, J.Y.; Qin, J.; Jin, T.; Xu, J.M. Long noncoding RNA AFAP1-AS1 indicates a poor prognosis of hepatocellular carcinoma and promotes cell proliferation and invasion via upregulation of the RHOA/RAC2 signaling. *Int. J. Oncol.* **2016**, *48*, 1590–1598, doi:10.3892/ijo.2016.3385.
57. Yuan, J.H.; Yang, F.; Wang, F.; Ma, J.Z.; Guo, Y.J.; Tao, Q.F.; Liu, F.; Pan, W.; Wang, T.T.; Zhou, C.C.; et al. A long noncoding RNA activated by tgf-beta promotes the invasion-metastasis cascade in hepatocellular carcinoma. *Cancer Cell* **2014**, *25*, 666–681, doi:10.1016/j.ccr.2014.03.010.
58. Chen, F.; Li, Y.; Feng, Y.; He, X.; Wang, L. Evaluation of antimetastatic effect of LncRNA-ATB sirna delivered using ultrasound-targeted microbubble destruction. *DNA Cell Biol.* **2016**, *35*, 393–397, doi:10.1089/dna.2016.3254.
59. Li, Y.; Ye, Y.; Chen, H. Astragaloside iv inhibits cell migration and viability of hepatocellular carcinoma cells via suppressing long noncoding RNA ATB. *Biomed. Pharmacother.* **2018**, *99*, 134–141, doi:10.1016/j.biopha.2017.12.108.

60. Wang, T.H.; Wu, C.H.; Yeh, C.T.; Su, S.C.; Hsia, S.M.; Liang, K.H.; Chen, C.C.; Hsueh, C.; Chen, C.Y. Melatonin suppresses hepatocellular carcinoma progression via lncRNA-CPS1-it-mediated HIF-1α inactivation. *Oncotarget* **2017**, *8*, 82280–82293, doi:10.18632/oncotarget.19316.
61. Wang, T.H.; Yu, C.C.; Lin, Y.S.; Chen, T.C.; Yeh, C.T.; Liang, K.H.; Shieh, T.M.; Chen, C.Y.; Hsueh, C. Long noncoding RNA CPS1-IT1 suppresses the metastasis of hepatocellular carcinoma by regulating HIF-1alpha activity and inhibiting epithelial-mesenchymal transition. *Oncotarget* **2016**, *7*, 43588–43603, doi:10.18632/oncotarget.9635.
62. Sui, C.J.; Zhou, Y.M.; Shen, W.F.; Dai, B.H.; Lu, J.J.; Zhang, M.F.; Yang, J.M. Long noncoding RNA GIHCG promotes hepatocellular carcinoma progression through epigenetically regulating miR-200b/a/429. *J. Mol. Med. (Berl)* **2016**, *94*, 1281–1296, doi:10.1007/s00109-016-1442-z.
63. Wang, F.; Yuan, J.H.; Wang, S.B.; Yang, F.; Yuan, S.X.; Ye, C.; Yang, N.; Zhou, W.P.; Li, W.L.; Li, W.; et al. Oncofetal long noncoding RNA PVT1 promotes proliferation and stem cell-like property of hepatocellular carcinoma cells by stabilizing NOP2. *Hepatology* **2014**, *60*, 1278–1290, doi:10.1002/hep.27239.
64. Zhang, Q.; Matsuura, K.; Kleiner, D.E.; Zamboni, F.; Alter, H.J.; Farci, P. Analysis of long noncoding RNA expression in hepatocellular carcinoma of different viral etiology. *J. Transl. Med.* **2016**, *14*, 328, doi:10.1186/s12967-016-1085-4.
65. Yan, X.; Zhang, D.; Wu, W.; Wu, S.; Qian, J.; Hao, Y.; Yan, F.; Zhu, P.; Wu, J.; Huang, G.; et al. Mesenchymal stem cells promote hepatocarcinogenesis via lncRNA-MUF interaction with ANXA2 and miR-34a. *Cancer Res.* **2017**, doi:10.1158/0008-5472.CAN-17-1915.
66. Xu, Y.C.; Liang, C.J.; Zhang, D.X.; Li, G.Q.; Gao, X.; Fu, J.Z.; Xia, F.; Ji, J.J.; Zhang, L.J.; Li, G.M.; et al. LncSHRG promotes hepatocellular carcinoma progression by activating HES6. *Oncotarget* **2017**, *8*, 70630–70641, doi:10.18632/oncotarget.19906.
67. Chen, Z.Z.; Huang, L.; Wu, Y.H.; Zhai, W.J.; Zhu, P.P.; Gao, Y.F. Lncsox4 promotes the self-renewal of liver tumour-initiating cells through STAT3-mediated SOX4 expression. *Nat. Commun.* **2016**, *7*, 12598, doi:10.1038/ncomms12598.
68. Wang, Y.; He, L.; Du, Y.; Zhu, P.; Huang, G.; Luo, J.; Yan, X.; Ye, B.; Li, C.; Xia, P.; et al. The long noncoding RNA LNCTCF7 promotes self-renewal of human liver cancer stem cells through activation of wnt signaling. *Cell Stem Cell* **2015**, *16*, 413–425, doi:10.1016/j.stem.2015.03.003.
69. Wu, J.; Zhang, J.; Shen, B.; Yin, K.; Xu, J.; Gao, W.; Zhang, L. Long noncoding RNA lnctcf7, induced by il-6/stat3 transactivation, promotes hepatocellular carcinoma aggressiveness through epithelial-mesenchymal transition. *J. Exp. Clin. Cancer Res.* **2015**, *34*, 116, doi:10.1186/s13046-015-0229-3.
70. Zhang, S.G.; Li, Y.F.; Zhao, N.N.; Lai, C.C.; Cheng, S.J.; Yan, J.; Zhang, P.; Wang, Z.; Wang, X.L.; Yang, P.H. Decreased expression of long non-coding RNA loc728290 in human hepatocellular carcinoma. *Oncol. Lett.* **2017**, *14*, 4551–4556, doi:10.3892/ol.2017.6776.
71. Xu, J.H.; Chang, W.H.; Fu, H.W.; Shu, W.Q.; Yuan, T.; Chen, P. Upregulated long non-coding RNA LOC90784 promotes cell proliferation and invasion and is associated with poor clinical features in HCC. *Biochem. Biophys. Res. Commun.* **2017**, *490*, 920–926, doi:10.1016/j.bbrc.2017.06.141.
72. Huang, X.; Gao, Y.; Qin, J.; Lu, S. LncRNA-MIAT promoted proliferation and invasion of HCC cells via sponging miR-214. *Am. J. Physiol. Gastrointest. Liver Physiol.* **2017**, doi:10.1152/ajpgi.00242.2017.
73. Nie, F.Q.; Zhu, Q.; Xu, T.P.; Zou, Y.F.; Xie, M.; Sun, M.; Xia, R.; Lu, K.H. Long non-coding RNA mvih indicates a poor prognosis for non-small cell lung cancer and promotes cell proliferation and invasion. *Tumour Biol.* **2014**, *35*, 7587–7594, doi:10.1007/s13277-014-2009-7.
74. Yuan, S.X.; Yang, F.; Yang, Y.; Tao, Q.F.; Zhang, J.; Huang, G.; Yang, Y.; Wang, R.Y.; Yang, S.; Huo, X.S.; et al. Long noncoding RNA associated with microvascular invasion in hepatocellular carcinoma promotes angiogenesis and serves as a predictor for hepatocellular carcinoma patients' poor recurrence-free survival after hepatectomy. *Hepatology* **2012**, *56*, 2231–2241, doi:10.1002/hep.25895.
75. Wang, L.; Guo, Z.Y.; Zhang, R.; Xin, B.; Chen, R.; Zhao, J.; Wang, T.; Wen, W.H.; Jia, L.T.; Yao, L.B.; et al. Pseudogene OCT4-PG4 functions as a natural micro RNA sponge to regulate oct4 expression by competing for miR-145 in hepatocellular carcinoma. *Carcinogenesis* **2013**, *34*, 1773–1781, doi:10.1093/carcin/bgt139.
76. Peng, W.; Fan, H. Long non-coding RNA pandar correlates with poor prognosis and promotes tumorigenesis in hepatocellular carcinoma. *Biomed Pharmacother* **2015**, *72*, 113–118, doi:10.1016/j.biopha.2015.04.014.
77. Wen, J.; Xu, J.; Sun, Q.; Xing, C.; Yin, W. Upregulation of long non coding RNA PCAT-1 contributes to cell proliferation, migration and apoptosis in hepatocellular carcinoma. *Mol. Med. Rep.* **2016**, *13*, 4481–4486, doi:10.3892/mmr.2016.5075.
78. Yan, T.H.; Yang, H.; Jiang, J.H.; Lu, S.W.; Peng, C.X.; Que, H.X.; Lu, W.L.; Mao, J.F. Prognostic significance of long non-coding RNA pcat-1 expression in human hepatocellular carcinoma. *Int. J. Clin. Exp. Pathol.* **2015**, *8*, 4126–4131.

79. Ren, Y.; Shang, J.; Li, J.; Liu, W.; Zhang, Z.; Yuan, J.; Yang, M. The long noncoding RNA PCAT-1 links the microrna miR-215 to oncogene crkl-mediated signaling in hepatocellular carcinoma. *J. Biol. Chem.* **2017**, *292*, 17939–17949, doi:10.1074/jbc.M116.773978.
80. Zhang, D.; Cao, J.; Zhong, Q.; Zeng, L.; Cai, C.; Lei, L.; Zhang, W.; Liu, F. Long noncoding RNA pcat-1 promotes invasion and metastasis via the miR-129-5p-HMGB1 signaling pathway in hepatocellular carcinoma. *Biomed. Pharmacother.* **2017**, *95*, 1187–1193, doi:10.1016/j.biopha.2017.09.045.
81. Wang, Y.; Hu, Y.; Wu, G.; Yang, Y.; Tang, Y.; Zhang, W.; Wang, K.; Liu, Y.; Wang, X.; Li, T. Long noncoding RNA PCAT-14 induces proliferation and invasion by hepatocellular carcinoma cells by inducing methylation of miR-372. *Oncotarget* **2017**, *8*, 34429–34441, doi:10.18632/oncotarget.16260.
82. Yuan, S.X.; Tao, Q.F.; Wang, J.; Yang, F.; Liu, L.; Wang, L.L.; Zhang, J.; Yang, Y.; Liu, H.; Wang, F.; et al. Antisense long non-coding RNA PCNA-AS1 promotes tumor growth by regulating proliferating cell nuclear antigen in hepatocellular carcinoma. *Cancer Lett.* **2014**, *349*, 87–94, doi:10.1016/j.canlet.2014.03.029.
83. Yeh, M.M.; Boukhar, S.; Roberts, B.; Dasgupta, N.; Daoud, S.S. Genomic variants link to hepatitis c racial disparities. *Oncotarget* **2017**, *8*, 59455–59475, doi:10.18632/oncotarget.19755.
84. Kong, Y.; Zhang, L.; Huang, Y.; He, T.; Zhang, L.; Zhao, X.; Zhou, X.; Zhou, D.; Yan, Y.; Zhou, J.; et al. Pseudogene PDIA3P1 promotes cell proliferation, migration and invasion, and suppresses apoptosis in hepatocellular carcinoma by regulating the p53 pathway. *Cancer Lett.* **2017**, *407*, 76–83, doi:10.1016/j.canlet.2017.07.031.
85. Zhou, C.C.; Yang, F.; Yuan, S.X.; Ma, J.Z.; Liu, F.; Yuan, J.H.; Bi, F.R.; Lin, K.Y.; Yin, J.H.; Cao, G.W.; et al. Systemic genome screening identifies the outcome associated focal loss of long noncoding RNA pral in hepatocellular carcinoma. *Hepatology* **2016**, *63*, 850–863, doi:10.1002/hep.28393.
86. Chen, C.L.; Tseng, Y.W.; Wu, J.C.; Chen, G.Y.; Lin, K.C.; Hwang, S.M.; Hu, Y.C. Suppression of hepatocellular carcinoma by baculovirus-mediated expression of long non-coding RNA ptenp1 and microrna regulation. *Biomaterials* **2015**, *44*, 71–81, doi:10.1016/j.biomaterials.2014.12.023.
87. Qian, Y.Y.; Li, K.; Liu, Q.Y.; Liu, Z.S. Long non-coding RNA PTENP1 interacts with miR-193a-3p to suppress cell migration and invasion through the pten pathway in hepatocellular carcinoma. *Oncotarget* **2017**, *8*, 107859–107869, doi:10.18632/oncotarget.22305.
88. Cui, H.; Zhang, Y.; Zhang, Q.; Chen, W.; Zhao, H.; Liang, J. A comprehensive genome-wide analysis of long noncoding RNA expression profile in hepatocellular carcinoma. *Cancer Med.* **2017**, doi:10.1002/cam4.1180.
89. Wang, F.; Yuan, J.H.; Wang, S.B.; Yang, F.; Yuan, S.X.; Ye, C.; Yang, N.; Zhou, W.P.; Li, W.L.; Li, W.; et al. Oncofetal long noncoding RNA PVT1 promotes proliferation and stem cell-like property of hepatocellular carcinoma cells by stabilizing NOP2. *Hepatology* **2014**, *60*, 1278–1290, doi:10.1002/hep.27239.
90. Gou, X.; Zhao, X.; Wang, Z. Long noncoding RNA pvt1 promotes hepatocellular carcinoma progression through regulating miR-214. *Cancer Biomark.* **2017**, doi:10.3233/CBM-170331.
91. Ding, C.; Yang, Z.; Lv, Z.; Du, C.; Xiao, H.; Peng, C.; Cheng, S.; Xie, H.; Zhou, L.; Wu, J.; et al. Long non-coding RNA PVT1 is associated with tumor progression and predicts recurrence in hepatocellular carcinoma patients. *Oncol. Lett.* **2015**, *9*, 955–963, doi:10.3892/ol.2014.2730.
92. Yu, J.; Han, J.; Zhang, J.; Li, G.; Liu, H.; Cui, X.; Xu, Y.; Li, T.; Liu, J.; Wang, C. The long noncoding RNAs PVT1 and uc002mbe.2 in sera provide a new supplementary method for hepatocellular carcinoma diagnosis. *Medicine (Baltimore)* **2016**, *95*, e4436, doi:10.1097/MD.0000000000004436.
93. Liu, J.; Lu, C.; Xiao, M.; Jiang, F.; Qu, L.; Ni, R. Long non-coding RNA SNHG20 predicts a poor prognosis for HCC and promotes cell invasion by regulating the epithelial-to-mesenchymal transition. *Biomed. Pharmacother.* **2017**, *89*, 857–863, doi:10.1016/j.biopha.2017.01.011.
94. Zhang, D.; Cao, C.; Liu, L.; Wu, D. Up-regulation of lncrna SNHG20 predicts poor prognosis in hepatocellular carcinoma. *J. Cancer* **2016**, *7*, 608–617, doi:10.7150/jca.13822.
95. Birgani, M.T.; Hajjari, M.; Shahrisa, A.; Khoshnevisan, A.; Shoja, Z.; Motahari, P.; Farhangi, B. Long non-coding RNA SNHG6 as a potential biomarker for hepatocellular carcinoma. *Pathol. Oncol. Res.* **2017**, doi:10.1007/s12253-017-0241-3.
96. Cao, C.; Zhang, T.; Zhang, D.; Xie, L.; Zou, X.; Lei, L.; Wu, D.; Liu, L. The long non-coding RNA, SNHG6-003, functions as a competing endogenous RNA to promote the progression of hepatocellular carcinoma. *Oncogene* **2017**, *36*, 1112–1122, doi:10.1038/onc.2016.278.
97. Yu, G.; Lin, J.; Liu, C.; Hou, K.; Liang, M.; Shi, B. Long non-coding RNA SPRY4-IT1 promotes development of hepatic cellular carcinoma by interacting with erralpha and predicts poor prognosis. *Sci. Rep.* **2017**, *7*, 17176, doi:10.1038/s41598-017-16781-9.

98. Zhou, M.; Zhang, X.Y.; Yu, X. Overexpression of the long non-coding RNA SPRY4-IT1 promotes tumor cell proliferation and invasion by activating EZH2 in hepatocellular carcinoma. *Biomed. Pharmacother.* **2017**, *85*, 348–354, doi:10.1016/j.biopha.2016.11.035.
99. Jing, W.; Gao, S.; Zhu, M.; Luo, P.; Jing, X.; Chai, H.; Tu, J. Potential diagnostic value of lncrna SPRY4-IT1 in hepatocellular carcinoma. *Oncol. Rep.* **2016**, *36*, 1085–1092, doi:10.3892/or.2016.4859.
100. Tian, F.; Xu, J.; Xue, F.; Guan, E.; Xu, X. Tincr expression is associated with unfavorable prognosis in patients with hepatocellular carcinoma. *Biosci. Rep.* **2017**, *37*, doi:10.1042/BSR20170301.
101. Bo, C.; Li, N.; Li, X.; Liang, X.; An, Y. Long noncoding RNA uc.338 promotes cell proliferation through association with bmi1 in hepatocellular carcinoma. *Hum. Cell* **2016**, *29*, 141–147, doi:10.1007/s13577-016-0140-z.
102. Braconi, C.; Valeri, N.; Kogure, T.; Gasparini, P.; Huang, N.; Nuovo, G.J.; Terracciano, L.; Croce, C.M.; Patel, T. Expression and functional role of a transcribed noncoding RNA with an ultraconserved element in hepatocellular carcinoma. *Proc. Natl. Acad. Sci. USA* **2011**, *108*, 786–791, doi:10.1073/pnas.1011098108.
103. Huang, M.D.; Chen, W.M.; Qi, F.Z.; Sun, M.; Xu, T.P.; Ma, P.; Shu, Y.Q. Long non-coding RNA TUG1 is up-regulated in hepatocellular carcinoma and promotes cell growth and apoptosis by epigenetically silencing of KLF2. *Mol. Cancer* **2015**, *14*, 165, doi:10.1186/s12943-015-0431-0.
104. Li, J.; Zhang, Q.; Fan, X.; Mo, W.; Dai, W.; Feng, J.; Wu, L.; Liu, T.; Li, S.; Xu, S.; et al. The long noncoding RNA TUG1 acts as a competing endogenous RNA to regulate the hedgehog pathway by targeting miR-132 in hepatocellular carcinoma. *Oncotarget* **2017**, *8*, 65932–65945, doi:10.18632/oncotarget.19582.
105. Lin, Y.H.; Wu, M.H.; Huang, Y.H.; Yeh, C.T.; Cheng, M.L.; Chi, H.C.; Tsai, C.Y.; Chung, I.H.; Chen, C.Y.; Lin, K.H. Taurine up-regulated gene 1 functions as a master regulator to coordinate glycolysis and metastasis in hepatocellular carcinoma. *Hepatology* **2018**, *67*, 188–203, doi:10.1002/hep.29462.
106. Cao, S.W.; Huang, J.L.; Chen, J.; Hu, Y.W.; Hu, X.M.; Ren, T.Y.; Zheng, S.H.; Lin, J.D.; Tang, J.; Zheng, L.; et al. Long non-coding RNA UBE2CP3 promotes tumor metastasis by inducing epithelial-mesenchymal transition in hepatocellular carcinoma. *Oncotarget* **2017**, *8*, 65370–65385, doi:10.18632/oncotarget.18524.
107. Zheng, Z.K.; Pang, C.; Yang, Y.; Duan, Q.; Zhang, J.; Liu, W.C. Serum long noncoding RNA urothelial carcinoma-associated 1: A novel biomarker for diagnosis and prognosis of hepatocellular carcinoma. *J. Int. Med. Res.* **2017**, *46*, 348–356, doi:10.1177/0300060517726441.
108. Xiao, J.N.; Yan, T.H.; Yu, R.M.; Gao, Y.; Zeng, W.L.; Lu, S.W.; Que, H.X.; Liu, Z.P.; Jiang, J.H. Long non-coding RNA UCA1 regulates the expression of SNAIL2 by miR-203 to promote hepatocellular carcinoma progression. *J. Cancer Res. Clin. Oncol.* **2017**, *143*, 981–990, doi:10.1007/s00432-017-2370-1.
109. Wang, F.; Ying, H.Q.; He, B.S.; Pan, Y.Q.; Deng, Q.W.; Sun, H.L.; Chen, J.; Liu, X.; Wang, S.K. Upregulated LNCRNA-UCA1 contributes to progression of hepatocellular carcinoma through inhibition of miR-216b and activation of FGFR1/ERK signaling pathway. *Oncotarget* **2015**, *6*, 7899–7917, doi:10.18632/oncotarget.3219.
110. Hu, J.J.; Song, W.; Zhang, S.D.; Shen, X.H.; Qiu, X.M.; Wu, H.Z.; Gong, P.H.; Lu, S.; Zhao, Z.J.; He, M.L.; et al. HBX-upregulated lncrna UCA1 promotes cell growth and tumorigenesis by recruiting EZH2 and repressing P27KIP1/CDK2 signaling. *Sci. Rep.* **2016**, *6*, 23521, doi:10.1038/srep23521.
111. Kamel, M.M.; Matboli, M.; Sallam, M.; Montasser, I.F.; Saad, A.S.; El-Tawdi, A.H.F. Investigation of long noncoding RNAs expression profile as potential serum biomarkers in patients with hepatocellular carcinoma. *Transl. Res.* **2016**, *168*, 134–145, doi:10.1016/j.trsl.2015, doi:10.002.
112. Lv, L.; Chen, G.; Zhou, J.; Li, J.; Gong, J. Wt1-as promotes cell apoptosis in hepatocellular carcinoma through down-regulating of wt1. *J. Exp. Clin. Cancer Res.* **2015**, *34*, 119, doi:10.1186/s13046-015-0233-7.
113. Li, T.; Xie, J.; Shen, C.; Cheng, D.; Shi, Y.; Wu, Z.; Deng, X.; Chen, H.; Shen, B.; Peng, C.; et al. Upregulation of long noncoding RNA ZEB1-AS1 promotes tumor metastasis and predicts poor prognosis in hepatocellular carcinoma. *Oncogene* **2016**, *35*, 1575–1584, doi:10.1038/onc.2015.223.
114. Li, T.; Xie, J.; Shen, C.; Cheng, D.; Shi, Y.; Wu, Z.; Deng, X.; Chen, H.; Shen, B.; Peng, C.; et al. Amplification of long noncoding RNA ZFAS1 promotes metastasis in hepatocellular carcinoma. *Cancer Res.* **2015**, *75*, 3181–3191, doi:10.1158/0008-5472.CAN-14-3721.
